# Heterozygous mutations affecting the protein kinase domain of *CDK13* cause a syndromic form of developmental delay and intellectual disability

**DOI:** 10.1136/jmedgenet-2017-104620

**Published:** 2017-10-11

**Authors:** Mark J Hamilton, Richard C Caswell, Natalie Canham, Trevor Cole, Helen V Firth, Nicola Foulds, Ketil Heimdal, Emma Hobson, Gunnar Houge, Shelagh Joss, Dhavendra Kumar, Anne Katrin Lampe, Isabelle Maystadt, Victoria McKay, Kay Metcalfe, Ruth Newbury-Ecob, Soo-Mi Park, Leema Robert, Cecilie F Rustad, Emma Wakeling, Andrew O M Wilkie, The Deciphering Developmental Disorders Study, Stephen R F Twigg, Mohnish Suri

**Affiliations:** 1 West of Scotland Genetics Service, Queen Elizabeth University Hospital, Glasgow, UK; 2 Nottingham Clinical Genetics Service, Nottingham University Hospitals NHS Trust, Nottingham, UK; 3 Institute of Biomedical and Clinical Science, University of Exeter Medical School, Exeter, Devon, UK; 4 North West Thames Regional Genetics Service, London North West Healthcare NHS Trust, Harrow, UK; 5 West Midlands Regional Genetics Service, Birmingham Women’s NHS Foundation Trust, Birmingham, UK; 6 East Anglian Regional Genetics Service, Cambridge University Hospitals NHS Trust, Cambridge, UK; 7 Wellcome Trust Sanger Institute, Hinxton, UK; 8 Wessex Clinical Genetics Service, Southampton University Hospitals NHS Trust, Southampton, UK; 9 Section of Clinical Genetics, Department of Medical Genetics, Oslo University Hospital, Oslo, Norway; 10 Yorkshire Regional Clinical Genetics Service, The Leeds Teaching Hospitals NHS Trust, Leeds, UK; 11 Center for Medical Genetics and Molecular Medicine, Haukeland University Hospital, Bergen, Norway; 12 Institute of Medical Genetics, University Hospital of Wales, Cardiff, UK; 13 South East of Scotland Clinical Genetic Service, Western General Hospital, Edinburgh, UK; 14 Centre de Génétique Humaine, Institut de Pathologie et de Génétique (IPG), Gosselies, Belgium; 15 Merseyside Genetics Service, Liverpool Women’s NHS Foundation Trust, Liverpool, UK; 16 Manchester Centre for Genomic Medicine, Division of Evolution and Genomic Sciences, St Mary’s Hospital, Central Manchester University Hospitals NHS Foundation Trust Manchester Academic Health Sciences Centre, School of Biological Sciences, University of Manchester, Manchester, UK; 17 Clinical Genetics Service, University Hospital Bristol NHS Foundation Trust, Bristol, UK; 18 South East Thames Regional Clinical Genetics Service, Guy’s and St Thomas’ NHS Foundation, London, UK; 19 Clinical Genetics Group, MRC Weatherall Institute of Molecular Medicine, University of Oxford, Oxford, UK

**Keywords:** cdk13, congenital heart defects, ohdo syndrome, protein kinases, exome sequencing

## Abstract

**Introduction:**

Recent evidence has emerged linking mutations in *CDK13* to syndromic congenital heart disease. We present here genetic and phenotypic data pertaining to 16 individuals with *CDK13* mutations.

**Methods:**

Patients were investigated by exome sequencing, having presented with developmental delay and additional features suggestive of a syndromic cause.

**Results:**

Our cohort comprised 16 individuals aged 4–16 years. All had developmental delay, including six with autism spectrum disorder. Common findings included feeding difficulties (15/16), structural cardiac anomalies (9/16), seizures (4/16) and abnormalities of the corpus callosum (4/11 patients who had undergone MRI). All had craniofacial dysmorphism, with common features including short, upslanting palpebral fissures, hypertelorism or telecanthus, medial epicanthic folds, low-set, posteriorly rotated ears and a small mouth with thin upper lip vermilion. Fifteen patients had predicted missense mutations, including five identical p.(Asn842Ser) substitutions and two p.(Gly717Arg) substitutions. One patient had a canonical splice acceptor site variant (c.2898–1G>A). All mutations were located within the protein kinase domain of CDK13. The affected amino acids are highly conserved, and in silico analyses including comparative protein modelling predict that they will interfere with protein function. The location of the missense mutations in a key catalytic domain suggests that they are likely to cause loss of catalytic activity but retention of cyclin K binding, resulting in a dominant negative mode of action. Although the splice-site mutation was predicted to produce a stable internally deleted protein, this was not supported by expression studies in lymphoblastoid cells. A loss of function contribution to the underlying pathological mechanism therefore cannot be excluded, and the clinical significance of this variant remains uncertain.

**Conclusions:**

These patients demonstrate that heterozygous, likely dominant negative mutations affecting the protein kinase domain of the *CDK13* gene result in a recognisable, syndromic form of intellectual disability, with or without congenital heart disease.

## Introduction

The *CDK13* gene codes for a member of the cyclin-dependent serine/threonine protein kinase (STK) family, which phosphorylate targets in partnership with a cyclin co-factor; in this case, cyclin K. Recent work has shown that CDK13 plays a role in converting transcribing RNA polymerase II (Pol II) from an initiating to an elongating form, by phosphorylation of key residues in the C-terminal domain of Pol II. The genetic targets of *CDK13* activity, determined by knockdown of *CDK13* expression, appear to be largely involved in processes connected to extracellular and growth signalling.^1^

Mutations in *CDK13* had not previously been linked to human disease, until recent analysis of exome sequencing data arising from the Deciphering Developmental Disorders (DDD) study.^2^ In subanalysis of 398 trios affected by syndromic congenital heart disease (S-CHD), in which the proband did not have a plausible de novo mutation in a known developmental disorder-associated or CHD-associated gene, a significant clustering of de novo mutations in *CDK13* was observed. This led to identification of seven individuals with S-CHD, all carrying predicted missense variants within the highly conserved protein kinase (PK) domain of *CDK13*, including four with identical c.2525A>G (p.Asn842Ser) mutations. All had a history of developmental delay and craniofacial dysmorphism, characterised by hypertelorism, upslanted palpebral fissures, wide nasal bridge and narrow mouth, with additional features including structural brain abnormalities, clinodactyly, joint hypermobility and skin changes.^3^

In this context, we present genetic and phenotypic data relating to 16 patients investigated for intellectual disability (ID) syndromes and found to carry de novo mutations in *CDK13*, including those described by Sifrim *et al*.^3^ We have undertaken in silico analysis of the amino acid substitutions identified, including comparative protein modelling of selected variants based on the recently reported crystal structure for the kinase domain of CDK13 to predict their effect on ligand binding and thus catalytic activity. Key clinical data pertaining to the affected individuals are also presented, highlighting recognisable features that serve as pointers to this diagnosis.

## Methods

### Patient selection and diagnostic methods

All patients were referred to their regional clinical genetics service due to developmental delay with additional features, suspected to have an underlying syndromic cause. Patients 1–10 and 13–16 were investigated by exome sequencing as part of the DDD study.^2^ Patient 11 had whole-exome sequencing performed by the Clinical Genetics Group at the MRC Weatherall Institute of Molecular Medicine, University of Oxford. Patient 12 was investigated by whole-exome sequencing at the Center for Medical Genetics and Molecular Medicine at Haukeland University Hospital in Bergen. The molecular methods and bioinformatic pipeline for exome analysis used in the DDD study are previously described.^4^ Similar methods were used for exome sequencing analysis in patients 11^5^ and 12.

### RNA analysis

RNA was extracted from a lymphoblastoid cell line (patient 16) using Trizol (Invitrogen) and an RNeasy kit (Qiagen). For analysis of nonsense mediated decay (NMD), lymphoblastoid cells were treated with cyclohexamide (50 ng/µL) or media alone for 4 hours prior to RNA extraction. DNA was removed by digestion with DNase I (Qiagen) and cDNA synthesised using RevertAid First Strand Synthesis Kit (Thermo) with oligo(dt). Amplification was carried out using primers designed to exons flanking exon 11 (primer sequences available on request) and products sequenced using BigDye Terminator V.3.1 (Applied Biosystems).

### Protein modelling

Predicted effects of CDK13 variants on protein structure were modelled using the recently reported crystal structure of the kinase domain of CDK13 in complex with cyclin K (PDB identifier 5efq).^1^ Sequences of wild-type and variant CDK13 were submitted to the Swiss-Model server (https://swissmodel.expasy.org/ 6) for modelling in automated mode. Effects of variants on the thermodynamic stability of CDK13 were analysed using the FoldX modelling suite.^7^ As the crystal structure 5efq contains two heterodimers of CDK13-cyclinK, FoldX analysis was performed on the full asymmetric unit, each CDK13-cyclinK heterodimer (chains A-B and C-D) and each monomer of CDK13 in isolation (chains A and C), and values averaged across the five sets of results. In all cases, input files were first repaired with the RepairPDB function of FoldX, and variants introduced using the BuildModel function. In addition to the 10 novel missense variants reported here, analysis was performed on all missense variants in the gnomAD database occurring at residues present in structure 5efq (CDK13 residues 695–775, 785–1025; n=47). Structures were visualised in SwissPdb-Viewer^6^ or PyMOL (PyMOL Molecular Graphics System V.1.8, Schrödinger).

## Results

### Clinical findings

Phenotype data are summarised in table 1, with more detailed information provided as online supplementary file 1. The cohort comprised 14 females and 2 males, with an average age of 8 years (range 3.5–16.8). All patients had developmental delay, which was considered to be mild in two cases. Fifteen patients were reported to have a degree of learning disability, and one had IQ in the low-normal range. Six patients had a diagnosis of autism spectrum disorder, and a further two displayed autistic traits or stereotypies. Pica was reported in two cases. Four were born preterm (defined as gestation <37 weeks), and birth weight was distributed throughout the normal range, only one being less than the second centile.

10.1136/jmedgenet-2017-104620.supp1 Supplementary file 1

**Table 1:** Summary of clinical data from 15 patients with mutations in CDK13

Patient	1*	2*	3	4	5	6*	7*	8*	9*	10*	11	12	13	14	15	16
DDD number	271894	262889	259007	261411	264961	258830	265645	265813	259460	270818	NA	331720	264613	270857	301509	271710
*CDK13* mutation	p.Gly714Arg	p.Gly717Arg	p.Gly717Arg (mosaic)	p.Val719Gly	p.Lys734Arg	p.Arg751Gln	p.Asn842Ser	p.Asn842Ser	p.Asn842Ser	p.Asn842Ser	p.Asn842Ser	p.Asn842Asp	p.Arg860Gln	p.Val874Leu	p.Asp896Asn	c.2898–1G>A VoUS
Sex	F	M	F	F	F	F	F	F	F	M	F	F	F	F	F	F
Age at review	8.3	7.0	16.8	14.0	4.4	12.7	11.0	4	4.8	8.2	4.7	10.0	8.3	8.4	8.2	3.5
Gestational age at birth	38/40	38/40	38/40	34/40	36/40	35/40	40/40	38/40	41/40	39/40	37/40	37/40	38/40	40/40	40/40	36/40
Birth weight (centile)	19th	1st	15th	14th	42nd	3rd	70th	9th	42nd	2nd to 9th	75th	2nd to 9th	55th	50th	75th to 91st	62nd
Current height (centile)	9th to 25thth	0.4th	0.4th to 2nd	5th	1st	4th	1st at age 7 years	0.4th	5th	9th to 25th	3rd	<0.4th	<2nd	25th	34th	99th
Current weight (centile)	48th	0.4th	91st to 98th	67th	27th	59th	34th at age 7 years	9th	9th	0.4th to 2nd	0.4th to 2nd	<0.4th	2nd to 9th	50th	86th	96th
Current OFC (centile)	1st	<0.4th	9th to 25th	11th	1st	16th	9th to 25th	<0.4th	8th	<0.4th	30th	<0.4th	25th	25th to 50th	49th	ND
Developmental delay	+	+	+	+	+	+	+	+	+	+	+	+	+	+	+	+
Intellectual disability	+	+	+	+	+	+	+	+	+	+	– (WNV-IQ 86)	+	+	+	+	+
Autism	+	ND	+	+	–	–	+	–	–	+	–	–	(autistic traits)	–	(stereotypies)	+
Seizures	–	–	–	–	–	–	+	+	(febrile convulsions)	+	–	–	–	+	–	–
Feeding difficulties	+	+	–	+	+	+	+	+	+	+	+	+	+	+	+	+
Facial dysmorphism	+	+	+	+	+	+	+	+	+	+	+	+	+	+	+	+
Curly hair	+	–	–	+	+	–	+	–	+	–	–	+	–	+	–	–
Structural heart anomaly	+	+	–	–	–	+	+	+	+	+	+	+	–	–	–	–
Structural brain abnormality	+	+	–	–	ND	–	PVL	–	ND	+	ND	+	–	–	ND	ND
Digital anomalies	+	–	+	–	+	+	+	+	+	+	+	+	ND	+	+	+
Additional features	Microdontia, poor three-dimensional vision, overheats easily Glabellar haemangioma at 14 months	Nasal speech, sacral dimple, circumferential skin folds, recurrent mouth ulcers, required grommets	Obesity, circumferential skin folds, mild unilateral hearing loss	Sacral dimple, delayed osseous maturation, anal stenosis, recurrent gastrointestinal infections, sensorineural hearing impairment, lacrimal duct atresia, oligodontia	Pica. glabellar haemangioma, wide labial opening with partially deficient hymen, poor sleep, required grommets	Spinal hyperlordosis, truncal obesity	Spastic diplegia, scoliosis Panayiotopoulos syndrome	None	Pica. central sleep apnoea requiring oxygen	Poor sleep	Left congenital torticollis, glabellar haemangioma, sacral dimple, recurrent ear and upper respiratory tract infections	Metopic synostosis, congenital diaphragmatic hernia, choledochal cyst, recurrent respiratory infections	Low IgA and IgM	Glabellar haemangioma, painful dystonic spasms	Oligodontia with some small peg-shaped teeth, skin-picking behaviours	None

*Patients previously described by SIfrim *et al*.^3^

DDD, Deciphering Developmental Disorders; ND, no data; PVL, periventricular leukomalacia; VoUS, variant of uncertain significance; WNV-IQ, Wechsler non-verbal IQ.

Seizures of variable character were reported in four patients, including absence, myoclonic and generalised tonic-clonic episodes. One further patient had a history of febrile convulsions in infancy. A structural brain abnormality was identified in 5 of 11 patients who had undergone MRI, including thin or hypoplastic corpus callosum (3/5), absent corpus callosum (1/5) and periventricular leukomalacia (1/5) (see online supplementary file 2). Presence of a structural brain abnormality was not consistently associated with history of seizures. Feeding difficulties were common, reported in 15 patients. These included slow feeding, and susceptibility to gastro-oesophageal reflux in infancy. Concerns about safety of swallowing are frequently reported by parents (Katja Kyd, CDK13 Support Group, personal communication), and four patients in our series eventually required long-term feeding via percutaneous gastrostomy. Five had significant constipation, and a further individual had a history of anal stenosis and recurrent gastrointestinal infection. Structural cardiac anomalies were reported in nine patients, including six with atrial septal defect (ASD), two with ventricular septal defect (VSD) and one with both ASD and VSD. Auditory problems were frequently reported, including two patients requiring grommets, one with recurrent ear and upper respiratory tract infections, one with mild unilateral sensorineural hearing loss and one with bilateral sensorineural high-frequency hearing impairment. Central sleep apnoea was reported in one case.

**Figure 1:**
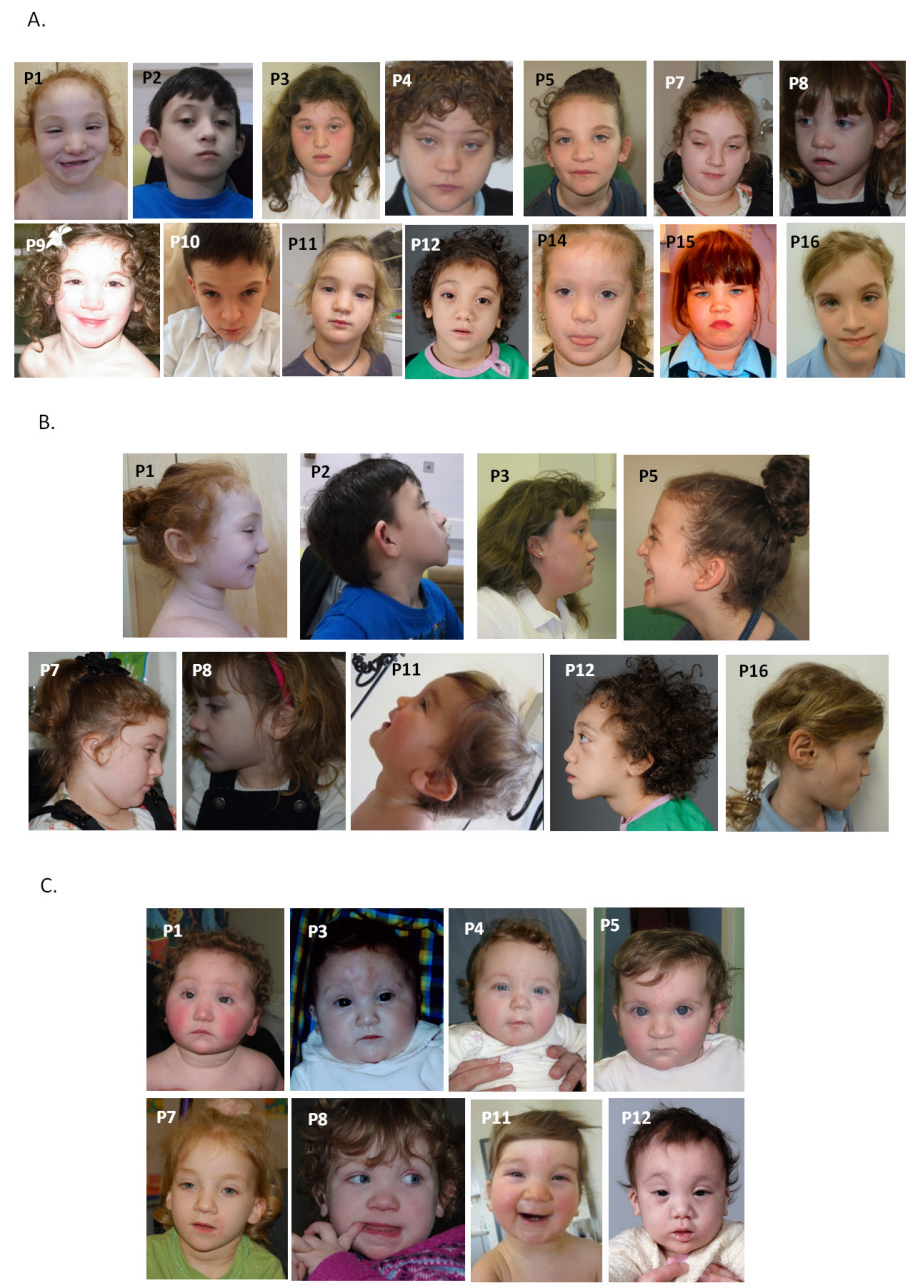
Anterior (A) and profile (B) facial photographs of 14 individuals with mutations in CDK13. Note hypertelorism or telecanthus, upslanting palpebral fissures with medial epicanthic folds, small mouth with thin upper lip and low set or posteriorly rotated ears. Clinical features overlapping those of Say-Barber-Biesecker-Young-Simpson variant of Ohdo syndrome are most evident in early childhood (C). Anterior photographs of patients 1 and 8 are reprinted by permission from Macmillan Publishers Ltd: *Nat Genet* 2016;48:1060-5.

All patients were reported to have craniofacial dysmorphic features (figure 1). Key findings included hypertelorism or telecanthus (9/16), upslanting palpebral fissures (8/16), medial epicanthic folds (8/16), small mouth (10/16) with thin upper lip (5/16) and low set and/or posteriorly rotated ears (10/16). The nose was relatively large with a full tip and alar flare in six patients, and glabellar haemangioma was present in infancy in four. Seven of the children were described as having curly hair. Facial resemblance was particularly striking in early childhood (figure 1C) with features overlapping those of the Say-Barber-Biesecker-Young-Simpson (SBBYS) variant of Ohdo syndrome.

Peripheral findings included joint hypermobility (2/16) and circumferential skin folds affecting the limbs (2/16). Minor digital anomalies were also common, including fifth finger clinodactyly (8/16) and prominent fetal finger pads (3/16).

### Mutations in CDK13

**Figure 2:**
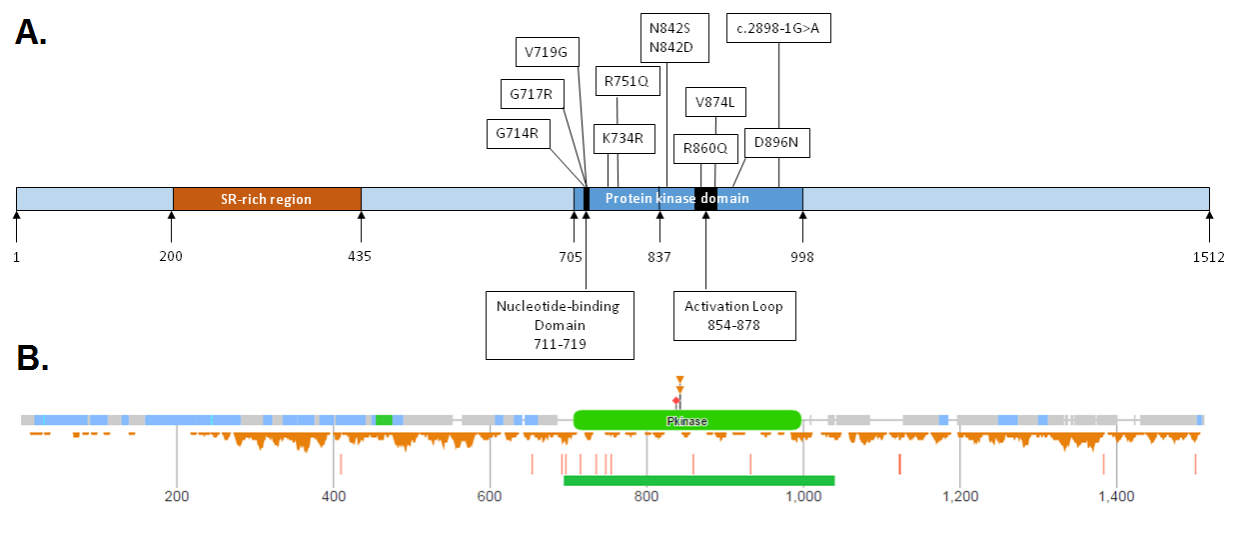
Schematic of CDK13 (UniProtKB Accession Q14004.1), demonstrating the position of the missense mutations and splice-site variant reported in patients. (B) Missense and loss-of-function variants in CDK13 reported on Exome Aggregation Consortium Server (figure courtesy of DECIPHER: decipher.sanger.ac.uk/). The missense variants are located below the polypeptide, whereas the loss of function variants are indicated as red vertical bars below the missense variants. The figure clearly shows the reduced number of missense variants reported in the protein kinase (Pkinase) domain of CDK13.

Fifteen patients were found to carry heterozygous mutations predicted to result in missense changes within the PK domain of *CDK13* (see online supplementary file 3 and figure 2A). These include four previously reported substitutions [p.Gly714Arg, p.Gly717Arg, p.Arg751Gln, p.Asn842Ser]. Five patients had identical p.(Asn842Ser) substitutions and two patients had the p.(Gly717Arg) substitution. One of the latter patients was mosaic for this mutation, with a higher mutation load in saliva compared with blood (data not shown). Previously unreported missense variants identified were p.Val719Gly, p.Lys734Arg, p.Asn842Asp, p.Arg860Gln, p.Val874Leu and p.Asp896Asn. One further patient was found to carry a canonical splice acceptor site mutation, c.2898–1G>A.

All missense substitutions affect highly conserved residues and are predicted to be pathogenic by in silico analysis. Moreover, all occur at positions of functional relevance in CDK13 (summarised in online supplementary file 3). In silico analysis of the canonical splice acceptor site mutation using a variety of splicing prediction algorithms suggested that it would disrupt splicing with likely skipping of exon 11.^8^ This would create a novel, in-frame splice junction between exons 10 and 12, and translation of the resulting mRNA would lead to synthesis of a truncated protein lacking amino acids 967–1010 of CDK13.

It was not possible to obtain RNA from primary cells from patient 16 to explore expression of the c.2898–1G>A variant; therefore, RT-PCR analysis was carried out on cDNA prepared from patient-derived lymphoblastoid cells (see online supplementary file 4). Surprisingly, this showed only the presence of the wild-type transcript, with no evidence of an exon 11-skipped product. Inhibition of NMD did reveal the presence of a mutant transcript, though the size difference from wild type was not sufficient to be consistent with skipping of exon 11. Instead, Sanger sequencing demonstrated use of a cryptic 3’ splice acceptor, just within exon 11; this new open reading frame terminated after five codons, which would be consistent with a mutant transcript subject to NMD.

### Variation and conservation in CDK13

The Exome Aggregation Consortium database^9^ calculates a constraint score, z, of 3.77 for missense variants in *CDK13*, indicating that there is general selection against coding variation in this gene. Inspection of the gnomAD database^8^ shows that no missense variants occur at positions 714, 717, 719, 734, 751, 842, 860, 874 or 896. Moreover, missense variants are generally rare across the PK domain in relation to the entire protein: a total of 573 unique variants have been reported at 480 positions across all of CDK13, at an average allele frequency of 2.86×10^–4^; however, for the 294-residue PK domain, only 41 variants at 39 positions have been reported, at an average frequency of 3.40×10^–5^ (figure 2B). Thus the PK domain is under greater selective pressure against variation, increasing the prior probability that any missense variant will be deleterious. Interestingly however, the probability of loss-of-function intolerance in CDK13 is only 0.75, and at the time of writing the gnomAD database lists 11 high-confidence loss of function variants which introduce stop codons in exons 1–13 (nine stop gain variants, two frameshifting indels). It is unlikely therefore that the phenotype observed in the patients described here results from a simple loss of function of CDK13, but suggests rather that a dominant negative mechanism is largely responsible.

To gain insight into the effect of the novel missense variants, amino acid conservation was explored using the ConSurf server (http://consurf.tau.ac.il/).^10^ This tool searches protein databases for related sequences, representing orthologues, paralogues and other homologous sequences, then uses these to compile a multiple sequence alignment (MSA). Positions of evolutionary conservation are indicated by a high ConSurf grade (range=1, low, to 9, high conservation) and low amino acid variety at a given position of interest. Analysis of residues 694–1039 of CDK13 (ie, the PK domain plus flanking residues) was carried out, yielding a total of 193 unique hits, of which the top-scoring 150 were used to build the MSA. Many of these proteins are, as yet, uncharacterised, but where names have been assigned these were overwhelmingly other cyclin-dependent kinases, mostly orthologues of *CDK12* and *CDK13* (data not shown). The ConSurf grades and amino acid variety at positions of the variants identified are included in online supplementary file 3.

Since all variants occur within the PK domain of CDK13, the PROSITE database (http://prosite.expasy.org/)^11^ was inspected for particular conservation within other PK family members. PROSITE entry PS50011 (PK domain profile) contains 4175 true positive hits, including CDK13, from which a sequence logo representing amino acid frequency at each position can be constructed (see online supplementary file 5). Inspection of the entry for CDK13 shows that residues 714, 717, 719, 734, 751, 842, 860, 874 and 896 lie at positions 10, 13, 15, 30, 47, 129, 147, 160 and 182 respectively of the PS50011 motif. Residues Gly714, Gly717 and Val719 all lie within the nucleotide-binding region of CDK13 (see figure 2); these residues are all strongly conserved within the PS50011 logo, as is the lysine at position 30 (CDK13 Lys734), which in the UniProtKB entry for CDK13 (Q14004; http://www.uniprot.org/uniprot/Q14004) is annotated as an ATP binding site. Furthermore, while some variation does occur at position 13, alanine and serine are the only commonly occurring amino acids other than glycine, and the variant amino acid arginine is not observed at significant frequency. Similarly at position 129, asparagine is almost invariant, while there is no significant occurrence of the variant serine residue. In contrast, there is greater amino acid variety at positions 47, 147 and 160, although in all cases the wild-type CDK13 residues (Arg751, Arg860 and Val874, respectively) are the most frequent; in addition, the variant residues at these positions (glutamine at positions 47 and 147, leucine at 160) do not occur at significant frequency at these positions in PK. Residue Asp896 lies outside known functional domains, but is strongly conserved in the PS50011 motif and is invariant in Pol II-associated cyclin-dependent kinases (CDKs 7, 8, 9, 12 and 13) and therefore likely plays an important structural role in CDK13 and other PKs.

10.1136/jmedgenet-2017-104620.supp5 Supplementary file 5

Taken together, this variation and conservation data indicates a strong preference for the wild-type amino acids at all positions of variance: glycines 714 and 717, Val719, Lys734 and Asn842 and Asp896 appear to be important for kinase structure/function in general, while arginines 751 and 860, and Val874, which are all highly conserved in ConSurf alignments, may play a more specific role in CDK13 and closely related proteins. The lack of any variation in the general population also suggests intolerance of missense substitutions at these positions.

### Comparative protein modelling

**Figure 3:**
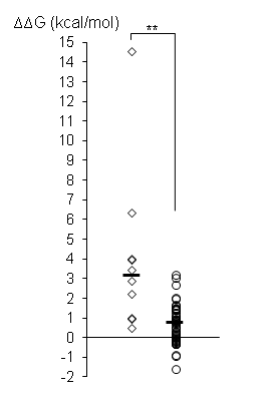
Effects of CDK13 missense substitutions on thermodynamic stability. Missense substitutions were introduced into the CDK13 structure 5efq using FoldX; results show average ∆∆G values for each variant calculated on all forms of CDK13 in 5efq (the asymmetric unit, two CDK13-cyclin K heterodimers and two CDK13 monomers in isolation). Data are shown for the 10 novel missense variants described in this report (rhomboids) and for 47 missense variants reported in the gnomAD database in residues present in the 5efq structure (circles); horizontal bars show the median value for each data set (novel variants: 3.19 kcal/mol; gnomAD variants, 0.76 kcal/mol); the two data sets were significantly different by two-tailed t-test (p<0.01, represented by **).

To investigate the effects of variants on CDK13 structure, and to help understand the molecular basis for pathogenicity and a putative dominant negative effect, we carried out comparative modelling of the missense variants based on the known crystal structure of the CDK13-cyclin K complex, 5efq. We also used the FoldX suite to evaluate the change in thermodynamic stability, ∆∆*G*, resulting from the various missense substitutions; these values are included in online supplementary file 3, and summarised in figure 3. Large positive changes in ∆∆*G* may be indicative of a loss of protein stability, and a recent comprehensive study of *MSH2* mutations in Lynch syndrome has shown a correlation between high ∆∆*G* values and loss of stability or reduced function.^12^ It should be noted however that not all variants adhered to this general rule, and it is possible that structural perturbations in one part of the protein, resulting in high ∆∆*G* values as calculated by FoldX, might be accommodated without causing loss of stability in other regions or domains. Furthermore, the use of thermodynamic calculations is only likely to help identify loss-of-function variants that have significant effects on protein structure, and is unlikely to be able to discriminate benign variants from those having position-specific effects resulting in altered function.

Several of the novel variants reported here had ∆∆*G* values which fell within the range observed for missense variants in the gnomAD database (−1.55 to +3.24 kcal/mol), and which fell below the value of 3 kcal/mol above which it is generally considered that substitutions might have significant deleterious effects on protein structure. It is unlikely therefore that these variants, namely Val719Arg, Lys734Arg, Arg751Gln, Arg860Gln and Val874Leu, cause significant loss of protein stability resulting in a general loss of function, implying that other mechanisms are responsible for the phenotype observed in patients carrying these variants.

**Figure 4:**
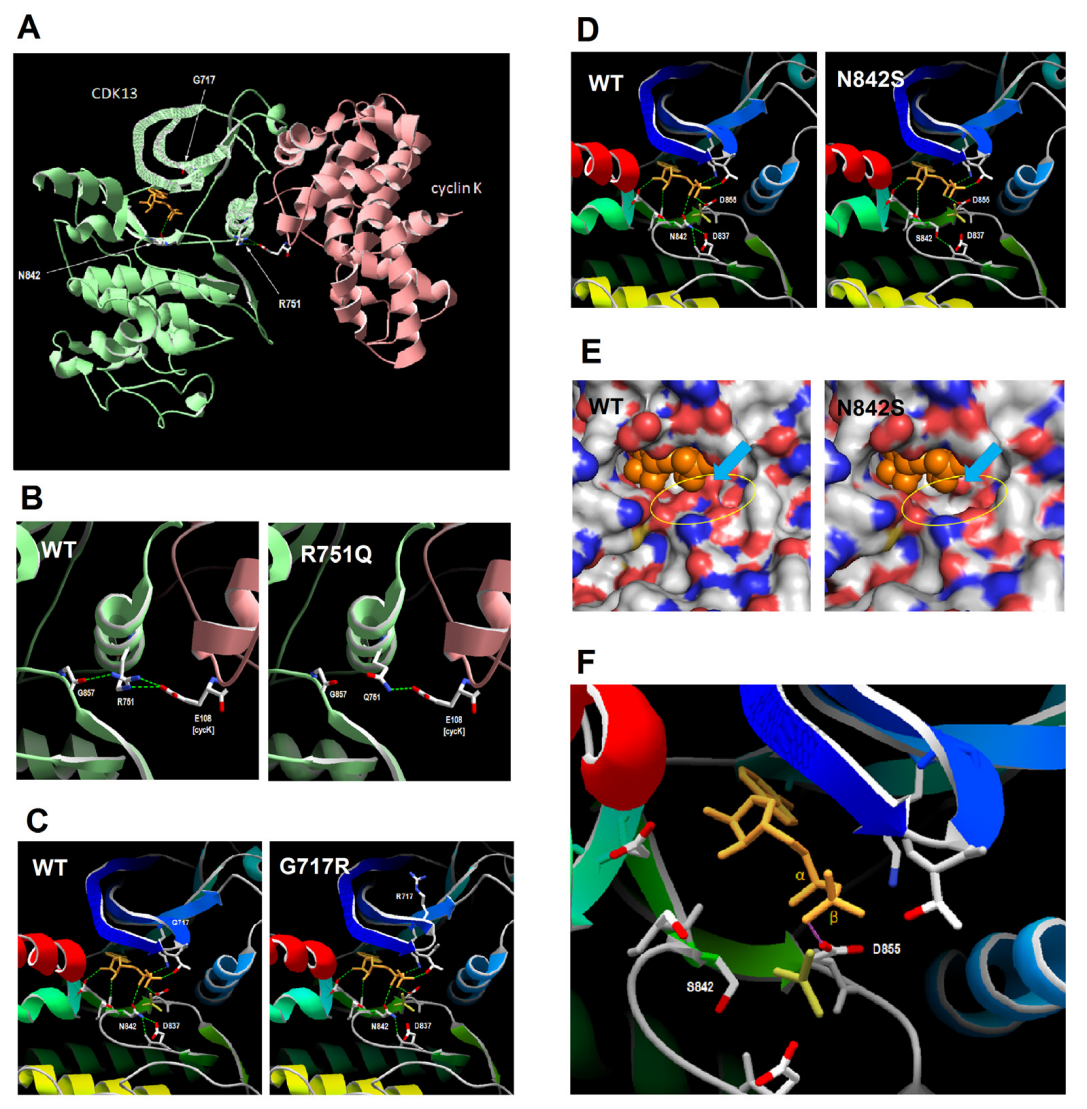
Comparative protein modelling of CDK13 variants. (A) Residues 694–1039 (kinase domain) of CDK13 were modelled on template 5efq; proteins are shown in ribbon format, with sidechains of selected residues and ligands (orange) shown in stick format; broken green lines indicate hydrogen bonds between selected groups. (B) Hydrogen bonding by CDK13 residue 751 in wild-type CDK13 and the Arg751Gln (R751Q) variant; CDK13 and cyclin K are shown in ribbon format, coloured green and pink, respectively. (C) Detail of ligand bonding in wild-type CDK13 and Gly717Arg (G717R) variant; the CDK13 backbone is shown in ribbon format, coloured from blue (N-terminal) to red (C-terminal); the ADP and AlF_3_ ligands are shown in stick format, coloured orange and dark yellow respectively; sidechains are shown for residues making H-bonds to ligands in wild-type CDK13, and for residue 717; note altered hydrogen bonding (green broken lines) between Asn842 (N842) and the active site residue Asp837 (D837), and the altered topology of the Gly717 (G717)-containing loop. (D) Modelling of the Asn842Ser variant initially returned structures with no bound ligand; therefore, the CDK13 Asn842Ser-ligand complex shown was manually assembled in Swiss-PDB Viewer and shows the potential H-bonds to the ligand; note loss of bonding by the variant residue Ser842 both to the ADP ligand and to the active site residue Asp837, and altered hydrogen bonding of Asp855 to the α-, rather than the β-phosphate group of ADP. (E) Detail of ligand binding pocket in wild-type and Asn842Ser (N842S) variant; the protein surface is coloured by CPK notation (red, oxygen; blue, nitrogen; yellow, sulphur; grey, carbon); the ADP ligand is shown as orange spheres, with the AlF_3_ ion omitted for clarity; the N842S structure shown is for the manually assembled CDK13 N842S-ligand complex (see previous figure); note loss of contact between base of pocket and the ligand (highlighted by yellow oval), and displacement of Asp855 (D855) sidechain towards ligand (blue arrow). (F) Detail of ligand position in the N842S variant; the CDK13 backbone is shown in ribbon format, coloured from blue (N-terminal) to red (C-terminal); the ADP and AlF_3_ ligands are shown in stick format, coloured orange and dark yellow, respectively; sidechains are shown for residues making bonds to ADP in wild-type CDK13, but hydrogen bonds have been omitted for clarity; Swiss-PDB Viewer was used to select residues making clashes with other groups, shown by the pink line; note the clash between the sidechain of D855 and the β-phosphate group of ADP; no such clashes were observed in wild-type CDK13 (not shown).

The results of molecular modelling of three selected variants (Gly717Arg, p.Arg751Gln and p.Asn842Ser) are shown in figure 4 and are discussed in detail below; a summary of analysis of the remaining missense variants is provided in online supplementary file 6. Arg751 lies at the interface with cyclin K, and in structure 5efq makes direct hydrogen bonds and a salt bridge (ie, electrostatic interaction) with cyclin K Glu108, and also a hydrogen bond to the backbone of Gly857 in the CDK13 activation loop. Asn842 lies on the lower surface of the ligand binding pocket, and in 5efq makes a direct hydrogen bond with the ADP ligand, while Gly717 lies in a glycine-rich loop at the upper surface of the ligand binding pocket.

The Arg751Gln variant results in loss of one hydrogen bond to cyclin K Glu108 (figure 4B) and of the salt bridge (not shown). While this might be expected to result in weaker binding affinity for cyclin K, it should be noted that, in the crystal structure of the CDK13-cyclin K complex, the protein–protein interface extends over a significant area and involves a total of 16 residues of CDK13 (see online supplementary file 7). As such it is likely that Arg751 makes only a minor contribution to the overall cyclin K binding affinity. Consistent with this, in silico analysis of the CDK13-cyclin K interaction using FoldX showed that the average binding energy for the two heterodimers present in PDB structure 5efq was −21.94 kcal/mol for the wild-type structure compared with −21.01 kcal/mol for the Arg751Gln variant. Therefore while the variant does cause a slight decrease in binding affinity, this is modest in terms of the overall interaction energy between the two proteins and suggests that the Arg751Gln variant will retain the ability to bind cyclin K. This in turn suggests that, by contacting both cyclin K Asp108 and CDK13 Gly857, the key function of Arg751 is to act as a molecular switch that transmits the effects of cyclin K binding through the activation loop to the active site of CDK13. In the Arg751Gln variant, the hydrogen bond to Gly857 is also lost; the likely outcome is that this variant exhibits only basal kinase activity and lacks sensitivity to cyclin K-dependent activation.

The Gly717Arg substitution results in altered topology of the glycine-rich loop at the upper surface of the ligand binding pocket (figure 4C), leading to altered hydrogen bonding within this region (not shown). Moreover, there are subtle but significant movements of other ligand-binding residues resulting in altered hydrogen bonding between Asn842 and Asp837. Since Asp837 constitutes the ‘active site’ residue of CDK13 (ie, in the transition state it forms a bond to the γ-phosphate group of ATP before this is transferred to the substrate; this state is mimicked in the 5efq structure by the AlF_3_ ion), altered topology and bonding at this residue is highly likely to result in reduced catalytic activity. It is also likely that, as the Gly717Arg substitution occurs in a glycine-rich region which shows a high degree of evolutionary conservation (see above), this variant has a more profound effect on protein folding in vivo than is apparent from comparative modelling in silico. Indeed, this variant exhibited the highest ∆∆*G* value in FoldX analysis (+14.59 kcal/mol), suggesting that there is likely to be some loss of protein stability at least in the vicinity of the ligand binding pocket. However, it should be noted that some MSH2 mutations with similar ∆∆*G* values retained nuclear localization and the ability to interact with MSH6,^12^ suggesting either that a high ∆∆*G* value alone cannot predict loss of stability with certainty, or that a local loss of stability can be tolerated without disrupting other protein functions.

Modelling of the wild-type CDK13 sequence, and that of the Arg751Gln and Gly717Arg variants in Swiss-Model, returned structures that included both the ADP and AlF_3_ ligands. In contrast, modelling of the Asn842Ser variant returned a structure that contained neither ligand, suggesting that alterations to the topology of the ligand binding pocket make binding unfavourable. For the purposes of investigating the possible effects of the Asn842Ser variant, a CDK13-ligand complex was manually assembled in Swiss-PDB Viewer, using the coordinates of the ADP ligand from the wild-type complex structure, and used to predict hydrogen bonding. This showed both loss of bonding to the ligand by residue 842, and aberrant bonding of Asp855 to the α-, rather than the β-phosphate group of ADP (figure 4D). Taken at face value, such changes would be expected to result in loss of catalytic activity, and in fact mutation of the equivalent residue, Asn130, in the male germ cell-associated kinase MAK has been shown to cause complete loss of kinase activity, resulting in retinitis pigmentosa.^13^ However, given that the results of modelling in Swiss-Model suggested ligand binding is unfavourable in the Asn842Ser variant, the structure of the ligand binding pocket was examined in more detail. Visualisation of the predicted molecular surface showed that the Asn842Ser substitution creates a cavity at the base of the ligand binding pocket (Figure 4), consistent with loss of direct bonding to the ligand by Asn842; this cavity could allow entry of water molecules which would disrupt protein–ligand interactions. Furthermore, there is a slight displacement of the sidechain of Asp855 towards the ligand (figure 4E, blue arrow), and this also is consistent with aberrant bonding of this residue to the ligand. Finally, prediction of residues making clashes (ie, steric hindrance) revealed a clash between the sidechain of Asp855 and the β-phosphate group of ADP (figure 4F); this indicates that both groups are attempting to occupy the same molecular space, and explains why construction of the CDK13-ligand complex was energetically unfavourable in Swiss-Model. Taken together, this indicates that the Asn842Ser variant not only compromises the ability of CDK13 to accommodate the ligand in its binding pocket, but that even should it do so the molecular interactions are unlikely to support catalytic activity. This is consistent with results of FoldX analysis for the Asn842Ser and Asn842Asp variants, which reported ∆∆*G* values of 4.05 and 6.36 kcal/mol, respectively, suggesting that the variants are likely to cause at least some local disruption of the structural domain.

Modelling was also undertaken of the internally deleted protein lacking residues 967–1010 that was originally predicted by in silico analysis^8^ of the c.2898–1G>A putative splice-site mutation. Modelling suggests this protein would be capable of folding into a tertiary structure retaining the ability to bind cyclin K (see online supplementary data S4). Although production of a stable internally deleted protein could not be supported by our expression studies in lymphoblastoid cells, we include these modelling results as it remains possible that factors such as tissue-specific differences in alternative splicing or expression levels may favour exon skipping in some cell types that was not observable in the blood-derived cells we tested. Since we did not have access to primary cells from other tissues from patient 16, it was not possible to explore this hypothesis further within the current study.

## Discussion

We report here 10 different missense mutations and 1 canonical splice-site mutation, all within the PK domain of CDK13 in sixteen individuals with a syndromic developmental delay phenotype. The missense mutations described occur at positions that are highly conserved in orthologues of *CDK13*, and the related *CDK12*, and in the case of Gly714, Gly717, Val719, Lys734, Asn842 and Asp896, in PK in general. Structural analysis reveals that all missense variants will cause changes to bonding and/or structure that are likely to lead to significant loss of catalytic activity, either as a result of perturbations to the structure of the active site and/or the activation loop, or by reducing the ability of CDK13 to respond to binding of its essential cyclin K partner. Notably, a number of mutations at equivalent residues in other PK have been observed to reduce or abolish kinase activity: in male germ cell-associated kinase (MAK), mutations at both Gly13 and Asn130, equivalent to CDK13 Gly714 and Asn842, respectively, have been shown to abolish kinase activity and cause retinitis pigmentosa,^13^ while mutation of RAF1 Thr491, equivalent to CDK13 Arg860, causes loss of activity resulting in Noonan syndrome type 5.^14^ Furthermore, mutation of the ATP binding residue, equivalent to CDK13 Lys734, has been reported to cause total loss of kinase activity in numerous proteins, including MAPK14 (at Lys53), Wee1-like PK (Lys328), EIF2AK2 (Lys296) and HIPK1 (Lys219). Interestingly, mutation of the equivalent residue in IκB kinase-β, Lys44, has been shown to result in a dominant negative effect of the mutant over the wild-type protein.^15^ Since IκB kinase-β exists in a complex with IκB kinase-α and the NF-κB-inducing kinase NIK, both of which are essential for its function, the dominant negative effect is presumably mediated either by sequestration of these proteins into an inactive complex, or by competition with active complexes for binding to the substrate.

Numerous loss-of-function variants in *CDK13* have been reported in the gnomAD database, and as the gnomAD dataset does not include individuals with severe paediatric disease, this indicates either that these variants do not result in a phenotype or cause a very mild phenotype that may be difficult to identify. In addition, two unique heterozygous deletions involving exon 2 have been reported in the Database of Genomic Variants (http://dgv.tcag.ca/dgv/app/home), in a cohort of 2026 reportedly healthy subjects.^16^ The Decipher database^17^ lists four deletions <4 Mb in length overlapping *CDK13*; from the limited phenotype data available, none is reported as having cardinal features of our cohort such as structural heart anomaly, corpus callosum abnormality or seizures. Taken together, this suggests that the strong phenotype we observe in patients described here cannot be explained by a simple loss of function, and therefore implies a dominant negative mechanism similar to that observed for the Lys44 mutation in IκB kinase-β, resulting in a more profound effect on CDK13 activity. The similarity of phenotype observed in our patient cohort further argues that this arises from a common molecular mechanism in all cases.

Molecular modelling shows that all missense variants reported here are expected to retain the ability to interact with cyclin K. Since cyclins are, by their nature, limiting factors governing CDK activity, the predicted ability of the CDK13 variants described here to bind cyclin K would be expected to exert a dominant negative effect by sequestering cyclin K into inactive complexes. Given the role of *CDK13* in regulation of genes involved in processes connected to extracellular and growth signalling, these variants represent very strong causative candidates for disorders of development, and the number of different variants described here provides overwhelming genetic evidence that mutations in *CDK13* are indeed responsible for the phenotype observed. In addition, as cyclin K is also an essential cofactor for CDK12 activity, it is possible that some of the features observed are in fact due to loss of activity of CDK12 in addition to that of CDK13. However, since no mutations have yet been reported in CDK12, it remains to be seen whether this is indeed the case.

There remains uncertainty regarding the significance of the c.2898–1G>A canonical splice-site variant. Clinical features of patient 16 are highly consistent with the other cases presented, including craniofacial features, fifth finger clinodactyly, autism spectrum disorder and a history of feeding difficulties. While in silico predictions suggested this variant would produce a stable, exon 11-skipped protein retaining the ability to bind cyclin K, we were unable to support this hypothesis by expression studies in patient-derived lymphoblastoid cells. There is a possibility that this result could reflect an artefact of different RNA expression in these cells compared with the affected tissues, such as brain, in vivo. Alternatively, we cannot exclude a contribution of a loss-of-function mechanism to the phenotype, or indeed that this variant is unrelated to the child’s presentation.

With respect to the remaining variants described here, it is possible that there is a spectrum of molecular effects which influences the severity of the phenotype observed. For example, the Lys734Arg variant, which by analogy with other kinases is expected to exhibit a total loss of kinase activity, also had the lowest ∆∆*G* value of the variants reported here (0.53 kcal/mol). It is likely therefore that the effects of this substitution will be confined to the active site and have little or no effect on global protein stability or the ability to bind cyclin K. As such this variant might be expected to exhibit a strong dominant negative effect, and in fact the patient carrying this variant displayed one of the more severe phenotypes in our cohort, including growth restriction, microcephaly and moderate to severe ID. Similarly, the two variants at Asn842 are also expected to cause total loss of kinase activity due to loss of ATP binding. Patients carrying these variants also showed a severe phenotype: all had a structural heart anomaly (6/6; 100% vs 3/10; 30% with other mutations), and head circumference less than the third centile (3/6; 50% vs 3/10; 30%), seizures (3/6; 50% vs 1/10; 10%) and corpus callosum abnormalities (2/6; 33% vs 2/10; 20%) were also more common in this group. The Asn842Ser and Asn842Asp substitutions had somewhat higher ∆∆*G* values than Lys734Arg (4.05 and 6.36 kcal/mol respectively), consistent with structural perturbation of the ligand binding pocket observed in molecular models. However, it is notable that a number of MSH2 variants with similar or higher ∆∆*G* values still retained nuclear localisation and MSH6 binding activity.^12^ This suggests that cyclin K binding may likewise be unaffected in these CDK13 variants, and the similarity of phenotype of these patients compared with that of the patient carrying the Lys734Arg variant argues that there is indeed a common molecular basis of disease. In contrast, the Arg751Gln variant, which is likely to retain basal kinase activity and which may also have slightly weakened cyclin K binding might be expected to exert a less severe dominant negative effect at the molecular level, although from the clinical data available it is not clear if the patient with this mutation (patient 6) has a milder phenotype compared with others in the cohort. The small number of cases presented here therefore provides tentative evidence of a genotype–phenotype relationship, but proof of this will require functional studies and identification of additional cases carrying different CDK13 variants, both of which lie beyond the current scope of this report.

The clinical data we present provide evidence for a recognisable craniofacial gestalt among children with *CDK13* mutations, characterised by short, upslanting palpebral fissures with telecanthus or hypertelorism, a small mouth with thin upper lip vermillion and low set or posteriorly rotated ears. This is particularly evident in early childhood but can be seen in older children as well. Other features that may alert the clinician to this diagnosis include feeding difficulties from infancy, presence of ASD or VSD, seizures, thin or absent corpus callosum, and fifth finger clinodactyly or prominent foetal pads. Interestingly, the SBBYS variant of Ohdo syndrome (OMIM 603736) was considered in the differential diagnosis of four of the reported cases. Since the original description by Ohdo *et al* of a kindred with ID, blepharophimosis, hypoplastic teeth and congenital heart disease,^18^ a heterogeneous range of Ohdo and Ohdo-like cases have been reported (reviewed in ref.^19^). With the exception of *KAT6B* mutations identified in the SBBYS variant,^20^ and *MED12* in an X-linked variant of Ohdo,^21^ the lack of known molecular causes has so far hampered more definitive classification of this broad group of blepharophimosis-ID syndromes. Identification of *CDK13* mutations in our cohort adds a further molecular cause of phenotypes within this spectrum, and therefore should be considered in the differential diagnosis of unresolved cases with Ohdo, Ohdo-like or blepharophimosis-ID syndromes.

Although further large case series would be required to develop detailed guidance for the medical care of children with mutations in the PK domain of *CDK13,* our data do highlight several issues to which the clinician should be vigilant. The high prevalence of feeding difficulties with onset in infancy would suggest a need for close monitoring of swallowing and nutrition, with early intervention to support feeding if necessary. Results also support screening for structural cardiac abnormalities in all children with this diagnosis. Clinicians and carers should be aware of an increased risk of seizures, which may be associated with structural brain abnormalities such as absent corpus callosum. The significance of recurrent ear infections or sensorineural hearing loss reported in a minority of cases is not clear, although clinicians may also wish to consider additional screening of hearing.

In summary, this study adds further evidence to support mutations in the kinase domain of *CDK13* as a cause of a clinically recognisable form of syndromic intellectual disability, with or without congenital heart disease, most likely by a novel dominant negative mechanism. Functional studies of the variants described are warranted to confirm in silico predictions, and larger case series are required to further delineate the clinical phenotype and guide medical management of affected individuals.

10.1136/jmedgenet-2017-104620.supp2 Supplementary file 2

10.1136/jmedgenet-2017-104620.supp3 Supplementary file 3

10.1136/jmedgenet-2017-104620.supp4 Supplementary file 4

10.1136/jmedgenet-2017-104620.supp6 Supplementary file 6

10.1136/jmedgenet-2017-104620.supp7 Supplementary file 7
