# Supplementary material for: Heterozygous mutations affecting the protein kinase domain of CDK13 cause a syndromic form of developmental delay and intellectual disability
Source: J Med Genet. 2017 Oct 11;55(1):28–38. doi: 10.1136/jmedgenet-2017-104620 (PMC5749303; doi:10.1136/jmedgenet-2017-104620)
Supplement: Supplementary file 1 [file jmedgenet-2017-104620supp001.pdf]

**Pt number (DDD number)**	**1** (271894)	**2** (262889)	**3** (259077)	**4** (261411)	**5** (264961)
**Age**	8.3	7	16.75	14	4.39
**Sex**	F	M	F	F	F
**Gestational age at birth**	38/40	38/40	38/40	34/40	36/40
**Birth weight (kg)**	2.636 (19th centile)	2 (1st centile)	2.58 (15th centile)	1.8 (14th centile)	2.5 (42nd centile)
**Birth height**				*	
**Birth OFC (cm)**	30.8 (2nd centile)			*	34 (92nd centile)
**Current weight (kg)**	27 (48th centile)	17.5 (0.4th centile)	73.4 (91st-98th centile)	54.1 (67th)	15.5 (27th centile)
**Current height (cm)**	123 (9th-25th centile)	108.5 (0.4th centile)	150.5 (0.4th-2nd centile)	148.8 (5th centile)	94 (1st centile)
**Current OFC**	50.5 (1st centile)	49 (< 0.4th centile)	54 (9th-25th centile)	51 (at chronological age 8.87 years; 11th centile)	47.5 (1st centile)
**Developmental delay**	Yes	Yes	speech and language delay	Yes	Yes
**ID**	Yes	Yes	Yes (moderate)	Yes	Yes (moderate/ severe)
**Autism**	Yes (mild)		Yes	Yes	No
**Seizures**	No	No	No	No	No
**Feeding difficulties**	Yes (gastrostomy fed from 4 months to 4 years)	Sensory-based feeding difficulties. He is feed orally as well as through his percutaneous gastrostomy	No	Yes (from infancy, severe gastro-oesophageal reflux).	Poor suck for few days at birth. GO reflux. NGT inserted at 4 months for 3-4 months.
**Eye problems**	Poor 3-dimensional vision	Divergent squint in his right eye (initially, bilateral squint)	Reading glasses	Lacrimal duct atresia	Astigmatism
**Hearing loss**	No	No. Grommet insertion February 2010	mild unilateral hearing loss	High frequency sensorineural hearing impairment	Bilateral grommets inserted at 3 years

Pt number (DDD number)	**1** (271894)	**2** (262889)	**3** (259077)	**4** (261411)	**5** (264961)
**Facial dysmorphism**	Hypertelorism, USPFs, wide nasal bridge, short philtrum, glabellar haemangioma noted at 14 months, now faded.	Arched eyebrows, USPFs, bilateral ptosis, low-set ears, anteverted ears, triangular nose, flat philtrum, small mouth, very thin upper lip	Short palpebral fissures, hypertelorism, USPFs, low-set ears, posteriorly-rotated ears, short philtrum, small mouth	Flat facial profile, epicanthic folds, long eyelashes, depressed nasal bridge, low set and posteriorly rotated ears, a very short nasal columella, long philtrum, thin upper lip, small mouth and tongue	Long eyelashes, telecanthus, epicanthic folds, low-set ears, short philtrum, wide nasal bridge, broad nasal tip, low posterior hairline, capillary haemangioma (faded) over nasal root when seen at 9/12
**Digital anomalies**	5th finger clinodactyly, extra crease 5th finger	No	Tapering fingers with 5th finger clinodactyly, radial deviation at PIP joint of both 4th fingers, fetal finger pads, broad big toes with hallux valgus	Prominent foetal finger pads, relatively short fingers, deep-set fingernails	Broad tips to fingers
**Limbs**	Extra skin crease on forearms and back of right thigh	Circumferential skin folds	Circumferential skin folds	Normal	No
**Palate**	Normal		Short uvula	Normal	No
**CVS**	ASD and mild pulmonary branch stenosis	Perimembranous VSD with pulmonary valve stenosis	Normal echo	Normal echo and ECG	No but never had an echo
**Genital anomalies**		Paraphimosis	None. Menarche aged 13.5 years	Not specified	Wide labial opening with partially deficient hymen
**Bowel problems**		Gastro-oesophageal reflux in infancy	Constipation	Anal stenosis, severe gastro-oesophageal reflux, eosinophilic colitis, recurrent GI infections	No
**Urinary problems**		No	No	Nil	No
**Skin**	Normal	Hypertrichosis over elbows, legs and back	possible sebaceous cyst over scalp	Nil	No

Pt number (DDD number)	**1** (271894)	**2** (262889)	**3** (259077)	**4** (261411)	**5** (264961)
**Spine**	Normal	No	Straight	Nil	No
**Neurological findings**		Hypotonia	None	Global developmental delay and intellectual disability	No
**MRI brain**	Severe hypoplasia of the corpus callosum with only the anterior genu present. Arnold-Chiari 1 variant	Underdeveloped corpus callosum	Normal	Normal (including pituitary)	MRI not done
**Array**	Normal	Normal	Normal	Normal	Not done
**CDK13 mutation**	p.Gly714Arg	p.Gly717Arg	pGly717Arg (mosaic; higher mutation load in saliva compared to blood)	p.Val719Gly	p.Lys734Arg
**Other features**	Microdontia	Nasal speech. Sacral dimple. Recurrent mouth ulcers, occasional nosebleeds	Obesity (BMI 33.2; >99.6th centile)	Sacral dimple, delayed ossification of hand bones of 9 months, borderline growth hormone deficiency, oligodontia, thick curly hair, obesity with onset of puberty	Poor sleep
**Previous diagnosis**	Ohdo-like	Ohdo syndrome	Ohdo syndrome	Kabuki, Angelman and Smith-Magenis considered	

**Pt number (DDD number)**	**6** (258830)	**7** (265645)	**8** (265813)	**9** (259460)	**10** (270818)
**Age**	12.68	11	4	4.84	8.16
**Sex**	F	F	F	F	M
**Gestational age at birth**	35/40	40/40	38/40	41/40	39/40
**Birth weight (kg)**	1.7 (3rd centile)	3.64 (70th centile)	2.5kg (9th centile)	3.31 (42nd centile)	2.552 (2nd-9th centile)
**Birth height**					
**Birth OFC (cm)**				34 cm (33rd centile)	2nd centile
**Current weight (kg)**	41 (59th centile)	22.5 (34th centile) at age 7	15.1 (9th centile)	10.06 (9th centile)	19.7 (0.4th-2nd centile)
**Current height (cm)**	136 (4th centile)	106.8 (1st centile) at age 7	95.8 (0.4th centiel)	81 cm (`5th centile)	121.5 (9th-25th centile)
**Current OFC**	53 (16th centile)	51.2 (10th centile); 53 cm on 17.11.15 (9th-25th centile) aged 11 years	46.7 (<0.4th)	47 (8th centile)	44 (<0.4th centile at 1.25 y)
**Developmental delay**	Yes	Yes	Yes, especially speech	Yes	Yes
**ID**	Yes	Yes	Yes (moderate)	Yes (moderate)	Yes
**Autism**	No	Yes	No	No	Yes
**Seizures**	No	History of absence seizures with normal EEG. Single generalised seizure. Diagnosis of autonomic seizures and cyclical vomiting.	yes absences, myoclonic, grand mal	Febrile seizures age 1, none since	Yes (absences, on Valproate)
**Feeding difficulties**	Neonatal period	NG fed for the first eight to nine months of life, then required gastrostomy.	Yes	Yes, velopharyngeal dysfunction, abnormal swallow	Very slow feeder at birth, irritable with feeds
**Eye problems**	Squint, myopia	Squint	No	No	No
**Hearing loss**		No	No	No	No

Pt number (DDD number)	**6** (258830)	**7** (265645)	**8** (265813)	**9** (259460)	**10** (270818)
**Facial dysmorphism**	Plagiocephaly, short palpebral fissures, ptosis, USPFs, small mouth with downturned corners	Highly-arched, thick eyebrows, USPFs, epicanthic folds, preauricular pit, hypertelorism, low-set ears, posteriorly-rotated ears, prominent nasal bridge, thin upper lip, small mouth, low posterior hairline	Brachycephaly, thick hair, low forehead, thick eyebrows, hypertelorism, telecanthus, epicanthic folds, low-set ears, overfolded helix, small mouth with downturned corners	Hypertelorism, USPFs, bulbous nose, small mouth, thin upper lip. Very curly hair, not in keeping with other family members.	Lots of head hair, DSPFs, abnormality of pinna, short nose, long philtrum, thin upper lip
**Digital anomalies**	Camptodactyly of 2nd-5th fingers	Hyperextensible finger joints, 5th finger clinodactyly	Tapering fingers, proxinal thumbs	5th finger clinodactyly, 2nd and 3rd toe clinodactyly	5th finger camptodactyly
**Limbs**				Joint hypermobility	Normal
**Palate**		No		Normal	Normal
**CVS**	ASD, VSD	VSD	mild pulmonary artery branch stenosis, ASD	ASD	Secundum ASD - tiny and haemodynamically insignificant
**Genital anomalies**				No	Normal
**Bowel problems**		Episodic vomiting, gastro-oesophageal reflux, constipation	Constipation	No	Constipation
**Urinary problems**				No	No
**Skin**	Dry skin		hypertrichosis	Normal	Normal

Pt number (DDD number)	**6** (258830)	**7** (265645)	**8** (265813)	**9** (259460)	**10** (270818)
**Spine**	Hyperlordosis	Scoliosis		Normal	Normal
**Neurological findings**		Spastic diplegia		Hypotonia	Motor tics
**MRI brain**	Normal	Periventricular leukomalacia	Normal	MRI not done	Absent corpus callosum
**Array**	Normal	Normal	Normal	Normal	Normal
**CDK13 mutation**	p.Arg751Gln	p.Asn842Ser	p.Asn842Ser	p.Asn842Ser	p.Asn842Ser
**Other features**	Truncal obesity	EEG normal, given diagnosis of Panayiotopoulos sydrome aged 5y		Pica. Central sleep apnoea requiring overnigh oxygen.	Only 2 hours sleep in 24 hours (on melatonin)
**Previous diagnosis**			CHARGE-like		Rubinstein-Taybi syndrome

Pt number (DDD number)	**11** (not in DDD)	**12** (331720)	**13** (264613)	**14** (270857)	**15** (301509)
**Age**	4.67	10	8.33	8.42	8.16
**Sex**	F	F	F	F	F
**Gestational age at birth**	37/40	37/40 weeks (cesarean section)	38/40	40/40	term
**Birth weight (kg)**	3.054 (75th centile)	2.484 (2nd-9th centile)	3.061 (55th centile)	3.4 (50th centile)	3.71 (75th to 91st centile)
**Birth height**	47				
**Birth OFC (cm)**	34 (75th-91st centile)	34 cm (75th-91st centile)			
**Current weight (kg)**	13.8 (0.4th-2nd centile)	17 kg (<0.4th centile)	20.5 (2nd-9th centile)	26.3 (50th centile)	33.6 (86th centile)
**Current height (cm)**	98.7 (3rd centile)	120 cm (<0.4th centile)	116.5 (<2nd centile)	127.5 (25th centile)	126.1 (34th centile)
**Current OFC**	49.2 (30th centile)	46 cm (<0.4th centile)	52 (25th centile)	53 (25th-50th centile)	51.6 (49th centile)
**Developmental delay**	Yes (mild) - walked at 19 months, mild speech delay	Yes	Yes	Yes	moderate global
**ID**	No (low normal range; non verbal Wechsler IQ: 86)	Yes (severe)	Yes	Yes	Currently 3 years behind at school. Gap increasing
**Autism**	No	No	No (autistic traits)	No	Not diagnosed, but has hand flapping ++ and motor steryotypies
**Seizures**	No	No	No	Generalised tonic-clonic and focal	
**Feeding difficulties**	Neonatal period	Yes (neonatal)	Infancy	Yes (tube fed from 2 months, gastrostomy fed from 11 months to 5 years)	Slow to handle mixed textures, but okay now
**Eye problems**	No	No (narrow lacrimal duct)	Squint, mild ptosis	No	Bilateral esotropia, especially when tired
**Hearing loss**	No	No	No	No	No hearing loss

Pt number (DDD number)	**11** (not in DDD)	**12** (331720)	**13** (264613)	**14** (270857)	**15** (301509)
**Facial dysmorphism**	Glabellar congenital oedema and haemangioma, prominent metopic suture, hypertelorism, USPFs, posteriorly rotated ears, wide nasal bridge, broad nasal tip, small mouth	Yes	Medial epicanthic folds, broad nasal bridge, small mouth	Glabellar haemangioma, short USPFs, telecanthus, medial epicanthic folds, broad nasal bridge, posteriorly rotated ears, small mouth.	Blepharophimosis, epicanthic folds, broad nasal tip, tented upper lip, posteriorly rotated ears
**Digital anomalies**	5th finger clinodactyly, deep palmar creases	Fetal pads and 5th finger clinodactyly		Fetal finger pads	Tapering digits, broad sandle gap in feet
**Limbs**	No problems	Narrow heel bilat		Joint hypermobility	Pes planus
**Palate**	No cleft but nasal speech (?VPD)	No	Normal	High-arched	Normal
**CVS**	Secundum ASD (operated)	ASD		None	No abnormality - has seen cardiology
**Genital anomalies**	No	No		No	No
**Bowel problems**	No	Loose stools before 2 yrs of age		Gastro-oesophageal reflux, constipation	No
**Urinary problems**	No	Urinary tract infection twice		Normal	No
**Skin**	No problem	No	Unusual wrinkled skin over palms, severe eczema	Normal	normal

Pt number (DDD number)	**11** (not in DDD)	**12** (331720)	**13** (264613)	**14** (270857)	**15** (301509)
**Spine**	sacral dimple (normal spinal USS)	No		Normal	Straight
**Neurological findings**	Normal	hypotonia	Hypotonia	Painful dystonic spasms	Hand flapping, steryotypies involving hand and feet
**MRI brain**	MRI not done	Thin corpus callosum	Normal	Normal	MRI not done
**Array**	Normal	2008: dup chr3: 2715059-3212741 (pat inherited - suspect benign) and dup chr3: 3562164-3833018 (pat inherited - suspect benign)	Normal	Normal	normal
**CDK13 mutation**	p.Asn842Ser	p.Asn842Asp	p.Arg860Gln	p.Val874Leu	p.Asp896Asn
**Other features**	Left congenital torticollis, recurrent ENT and URTS (<4 y)	metopic synostosis, congenital diaphragmatic hernia, choledochal cyst (type 4), recurrent respiratory infections	Low IgA and IgM levels, parental consanguinity, brother has homozygous MICU1 mutation with myopathy, movement disorder, developmental delay, autism and hypothyroidism.	Heterozygous for paternally-inherited p.Thr83Met variant in *TECTA* gene	had some small peg shaped teeth in primary dentition, plus oligodontia, secondary dentition normal. Has skin picking habit with scars on limbs++
**Previous diagnosis**		No diagnosis			Ohdo considered

**Pt number (DDD number)**	**16** (271710)
**Age**	3.54
**Sex**	F
**Gestational age at birth**	36/40 (twin pregnancy)
**Birth weight (kg)**	2.693 (62nd centile)
**Birth height**	
**Birth OFC (cm)**	
**Current weight (kg)**	14.32 (96th centile)
**Current height (cm)**	92.1 (99th centile)
**Current OFC**	
**Developmental delay**	Yes (mild)
**ID**	Yes
**Autism**	Yes (mild)
**Seizures**	No
**Feeding difficulties**	Yes
**Eye problems**	No
**Hearing loss**	

**Pt number (DDD number)**	**16** (271710)
**Facial dysmorphism**	Arched eyebrows, prominent forehead, deep-set eyes, hypertelorism, epicanthic folds, low-set ears, posteriorly-rotated ears, prominent ear crus
**Digital anomalies**	Mild 5th finger clinodactyly
**Limbs**	
**Palate**	
**CVS**	Normal
**Genital anomalies**	No
**Bowel problems**	Mild constipation
**Urinary problems**	No
**Skin**	Normal

**Pt number (DDD number)**	**16** (271710)
**Spine**	Normal
**Neurological findings**	Hypotonia
**MRI brain**	MRI not done
**Array**	Not done
**CDK13 mutation**	c.2898-1G>A
**Other features**	No other features
**Previous diagnosis**	Ohdo-like
