# Supplementary material for: Heterozygous mutations affecting the protein kinase domain of CDK13 cause a syndromic form of developmental delay and intellectual disability
Source: J Med Genet. 2017 Oct 11;55(1):28–38. doi: 10.1136/jmedgenet-2017-104620 (PMC5749303; doi:10.1136/jmedgenet-2017-104620)
Supplement: Supplementary file 5 [file jmedgenet-2017-104620supp005.pptx]

## Slide 1
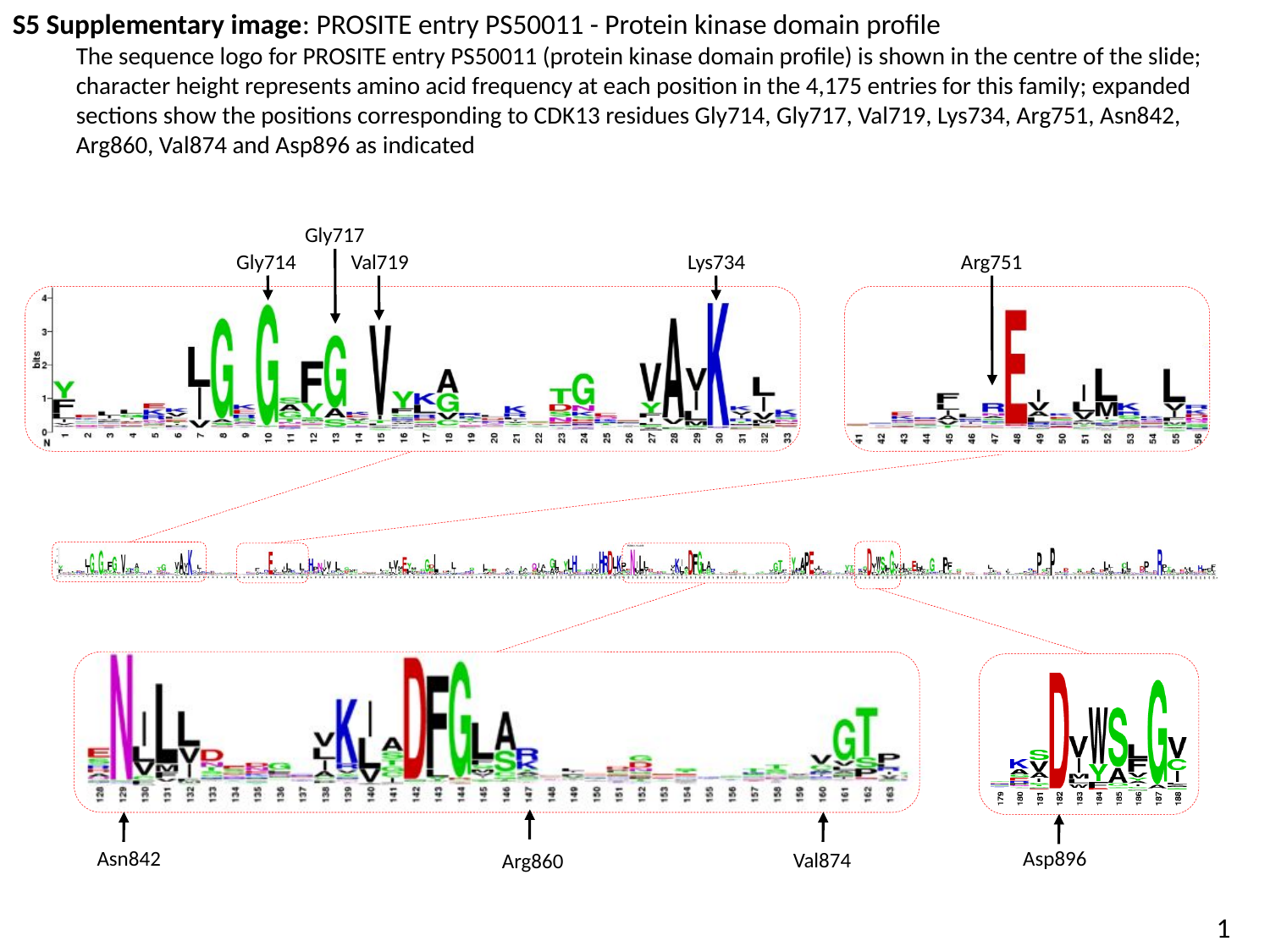

S5 Supplementary image: PROSITE entry PS50011 - Protein kinase domain profile
The sequence logo for PROSITE entry PS50011 (protein kinase domain profile) is shown in the centre of the slide; character height represents amino acid frequency at each position in the 4,175 entries for this family; expanded sections show the positions corresponding to CDK13 residues Gly714, Gly717, Val719, Lys734, Arg751, Asn842, Arg860, Val874 and Asp896 as indicated
Gly717
Gly714
Val719
Lys734
Arg751
Asn842
Val874
Arg860
Asp896
1
