# Supplementary material for: Heterozygous mutations affecting the protein kinase domain of CDK13 cause a syndromic form of developmental delay and intellectual disability
Source: J Med Genet. 2017 Oct 11;55(1):28–38. doi: 10.1136/jmedgenet-2017-104620 (PMC5749303; doi:10.1136/jmedgenet-2017-104620)
Supplement: Supplementary file 2 [file jmedgenet-2017-104620supp002.pptx]

## Slide 1
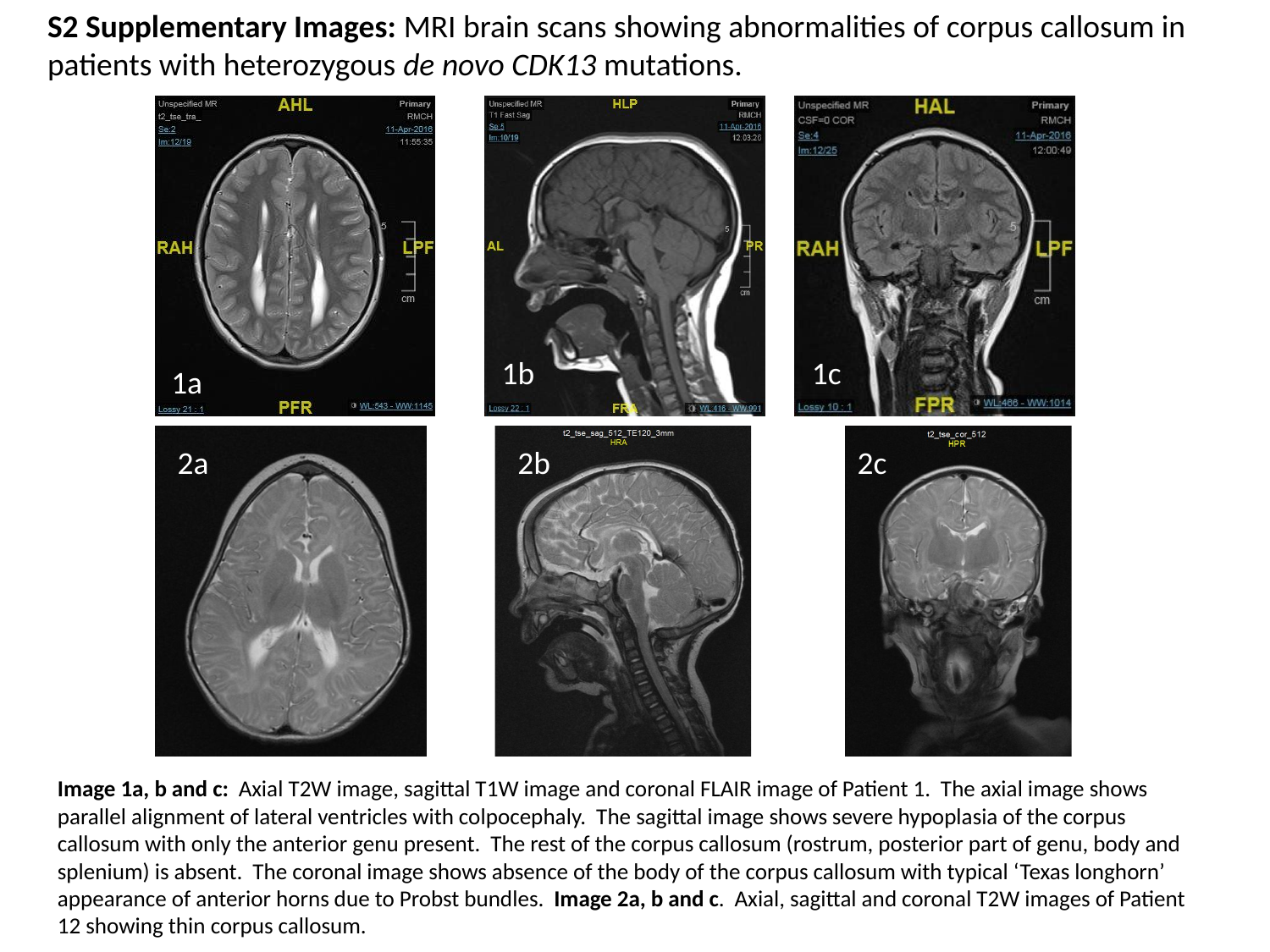

S2 Supplementary Images: MRI brain scans showing abnormalities of corpus callosum in patients with heterozygous de novo CDK13 mutations.
1b
1c
1a
2a
2b
2c
Image 1a, b and c: Axial T2W image, sagittal T1W image and coronal FLAIR image of Patient 1. The axial image shows parallel alignment of lateral ventricles with colpocephaly. The sagittal image shows severe hypoplasia of the corpus callosum with only the anterior genu present. The rest of the corpus callosum (rostrum, posterior part of genu, body and splenium) is absent. The coronal image shows absence of the body of the corpus callosum with typical ‘Texas longhorn’ appearance of anterior horns due to Probst bundles. Image 2a, b and c. Axial, sagittal and coronal T2W images of Patient 12 showing thin corpus callosum.
