# Supplementary material for: Heterozygous mutations affecting the protein kinase domain of CDK13 cause a syndromic form of developmental delay and intellectual disability
Source: J Med Genet. 2017 Oct 11;55(1):28–38. doi: 10.1136/jmedgenet-2017-104620 (PMC5749303; doi:10.1136/jmedgenet-2017-104620)
Supplement: Supplementary file 3 [file jmedgenet-2017-104620supp003.docx]

**^S3 supplementary table:^ *^In silico^* ^analysis of reported variants in^ *^CDK13^***

| c.DNA sequence change | c.2140G>C | c.2149G>A | c.2156T>G | c.2201A>G | c.2252G>A | c.2524A>G | c.2525A>G | c.2579G>A | c.2620G>T | c.2686G>A | c.2898-1G>A |
| --- | --- | --- | --- | --- | --- | --- | --- | --- | --- | --- | --- |
| *De novo* in reported cases? | Y | Y | Y | Y | Y | Y | Y | Y | Y | Y | NC |
| Predicted protein change^1^ | Gly714Arg | p.Gly717Arg | p.Val719Gly | p.Lys734Arg | p.Arg751Gln | p.Asn842Asp | p.Asn842Ser | p.Arg860Gln | p.Val874Leu | p.Asp896Asn | p.Ile967_Asp1010del |
| Consurf Grade^2^ | 9 | 9 | 9 | 9 | 9 | 9 | 9 | 9 | 9 | 9 | NA |
| Variety at this position (and % representation in MSA)^2^ | Gly (99.33%); other (0.67%) | Gly (98.00%); Ala (1.33%); other (0.67%) | Val (99.33%); 0ther (0.67%) | Lys (100.00%) | Arg (100.00%) | Asn (100.00%) | Asn (100.00%) | Arg (98.00%); Lys (2.00%) | Val (100.00%) | Asp (100.00%) | NA |
| Conserved amino acid? (species compared: chimpanzee, mouse, chicken, xenopus, zebrafish, drosophila, *C. elegans*)^3^ | Yes | Yes | Yes | Yes | Yes | Yes | Yes | Yes | Yes | Yes | NA |
| Variants at this position on gnomAD? ^4^ | No | No | No | No | No | No | No | No | No | No | No |
| Variant at this position on Exome Variant Server?^5^ | No | No | No | No | No | No | No | No | No | No | No |
| CADD^6^ | 32 | 34 | 28.7 | 27.8 | 34 | 25.6 | 24.4 | 35 | 33 | 34 | 26.9 |
| Polyphen2^7^ | Probably damaging (1) | Probably damaging (1) | Probably damaging (1) | Probably damaging (1) | Probably damaging (1) | Probably damaging (1) | Probably damaging (0.992) | Probably damaging (1) | Probably damaging (0.999) | Probably damaging (0.997) | NA |
| SIFT^8^ | Deleterious (0) | Deleterious (0) | Deleterious (0) | Deleterious (0.001) | Deleterious (0) | Deleterious (0) | Deleterious (0) | Deleterious (0) | Deleterious (0.01) | Deleterious (0) | NA |
| FoldX ∆∆*G* (kcal/mol) | +3.48 | +14.59 | +2.90 | +0.53 | +1.03 | +6.36 | +4.05 | +0.95 | +2.25 | +3.97 | NA |
| CDK13-cyclin K interaction energy (kcal/mol)^9^ | -21.95 | -21.91 | -21.95 | -21.95 | -21.01 | -21.96 | -21.96 | -21.76 | -21.93 | -21.93 | not determined |
| role in CDK13^10^ | Nucleotide binding domain | | | ADP/ATP binding | CycK binding | ADP/ATP binding | | Activation loop | | Structural | Nucleotide binding |

NC = inheritance not confirmed

^1^ Numbered according to the canonical 1,512 residue form of CDK13 encoded by Ensembl transcript ENST00000181839

^2^ The Consurf server. <http://consurf.tau.ac.il/>

^3^ Decipher Genome Browser: <https://decipher.sanger.ac.uk>

^4^ The Genome Aggregation Database (gnomAD): <http://gnomad.broadinstitute.org/>

^5^ <http://evs.gs.washington.edu/EVS/>

^6^ Combined Annotation Dependent Depletion (CADD). <http://cadd.gs.washington.edu/>

^7^ PolyPhen-2. <http://genetics.bwh.harvard.edu/pph2/>

^8^ Sorting Intolerant from Tolerant (SIFT). <http://sift.jcvi.org/>

^9^ average value for two CDK13-cyclin K dimers in crystal structure 5efq; the average value for wild type CDK13-cyclin K dimers was -21.94 kcal/mol

^10^ UniProt Knowledgebase (UniProtKB): <http://www.uniprot.org/> and PDBsum database: http://www.ebi.ac.uk/thornton-srv/databases/cgi-bin/pdbsum/GetPage.pl?pdbcode=index.html
