# Supplementary material for: Heterozygous mutations affecting the protein kinase domain of CDK13 cause a syndromic form of developmental delay and intellectual disability
Source: J Med Genet. 2017 Oct 11;55(1):28–38. doi: 10.1136/jmedgenet-2017-104620 (PMC5749303; doi:10.1136/jmedgenet-2017-104620)
Supplement: Supplementary file 4 [file jmedgenet-2017-104620supp004.pptx]

## Slide 1
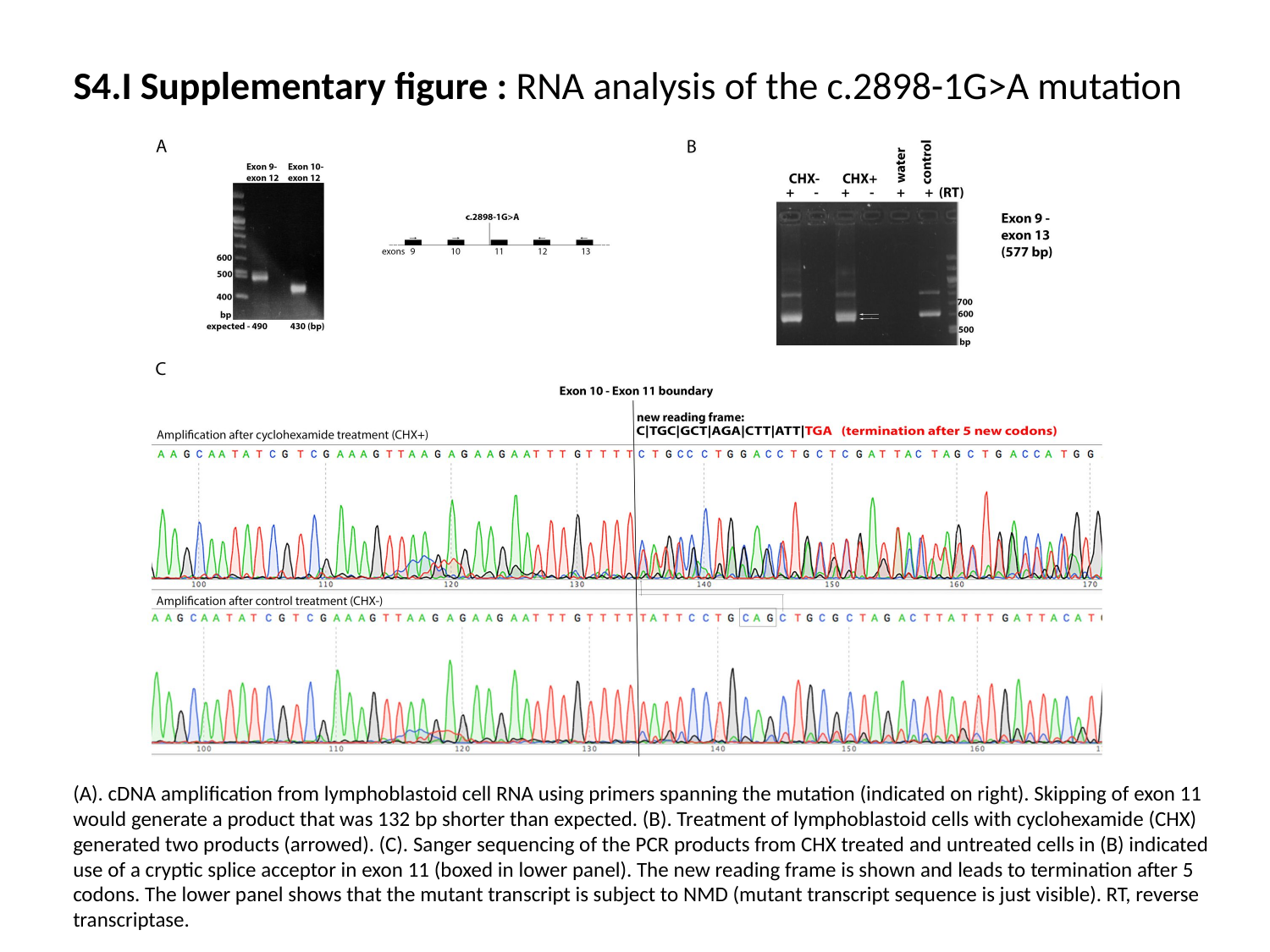

# S4.I Supplementary figure : RNA analysis of the c.2898-1G>A mutation
(A). cDNA amplification from lymphoblastoid cell RNA using primers spanning the mutation (indicated on right). Skipping of exon 11 would generate a product that was 132 bp shorter than expected. (B). Treatment of lymphoblastoid cells with cyclohexamide (CHX) generated two products (arrowed). (C). Sanger sequencing of the PCR products from CHX treated and untreated cells in (B) indicated use of a cryptic splice acceptor in exon 11 (boxed in lower panel). The new reading frame is shown and leads to termination after 5 codons. The lower panel shows that the mutant transcript is subject to NMD (mutant transcript sequence is just visible). RT, reverse transcriptase.

## Slide 2
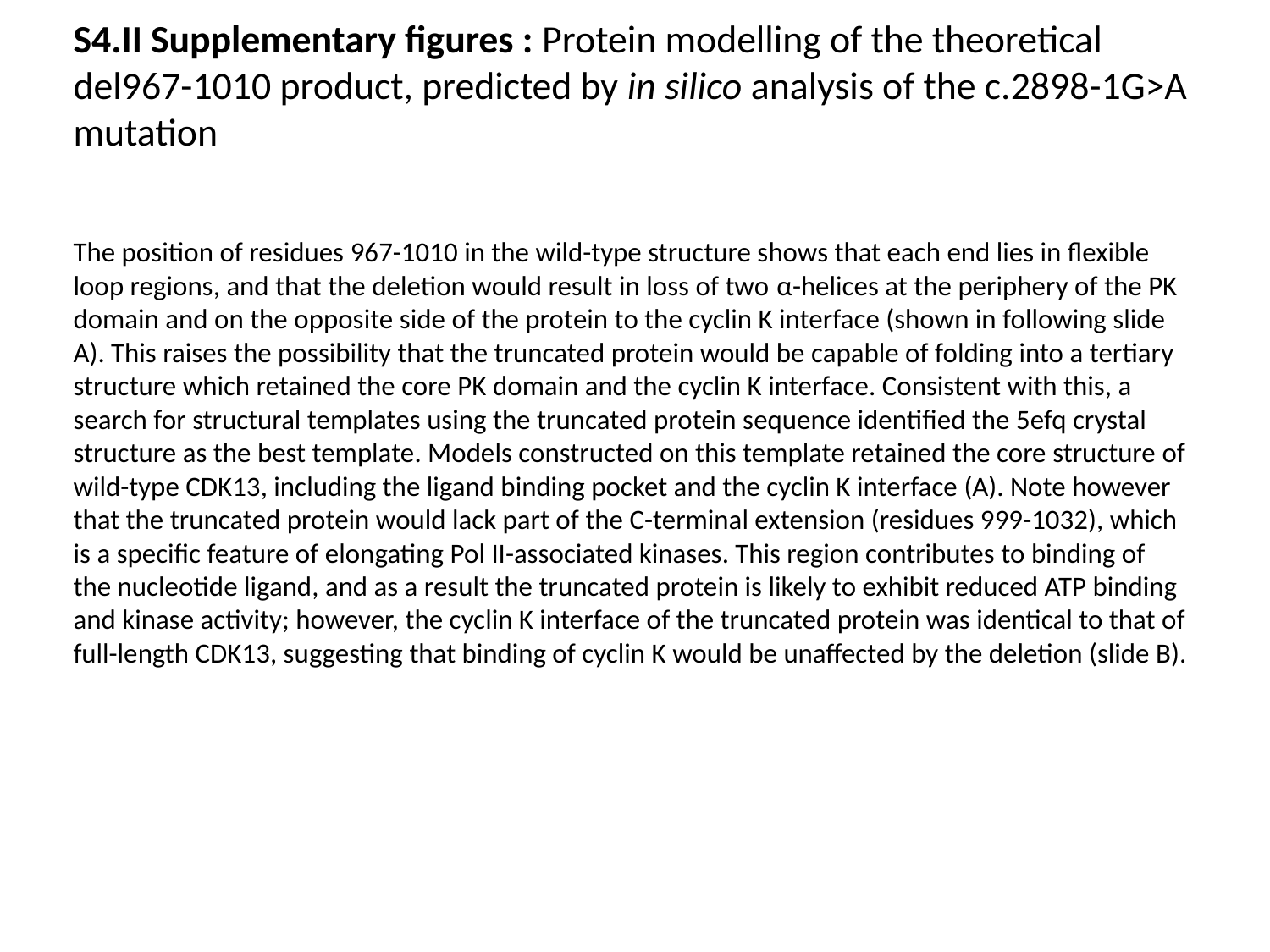

# S4.II Supplementary figures : Protein modelling of the theoretical del967-1010 product, predicted by in silico analysis of the c.2898-1G>A mutation
The position of residues 967-1010 in the wild-type structure shows that each end lies in flexible loop regions, and that the deletion would result in loss of two α-helices at the periphery of the PK domain and on the opposite side of the protein to the cyclin K interface (shown in following slide A). This raises the possibility that the truncated protein would be capable of folding into a tertiary structure which retained the core PK domain and the cyclin K interface. Consistent with this, a search for structural templates using the truncated protein sequence identified the 5efq crystal structure as the best template. Models constructed on this template retained the core structure of wild-type CDK13, including the ligand binding pocket and the cyclin K interface (A). Note however that the truncated protein would lack part of the C-terminal extension (residues 999-1032), which is a specific feature of elongating Pol II-associated kinases. This region contributes to binding of the nucleotide ligand, and as a result the truncated protein is likely to exhibit reduced ATP binding and kinase activity; however, the cyclin K interface of the truncated protein was identical to that of full-length CDK13, suggesting that binding of cyclin K would be unaffected by the deletion (slide B).

## Slide 3
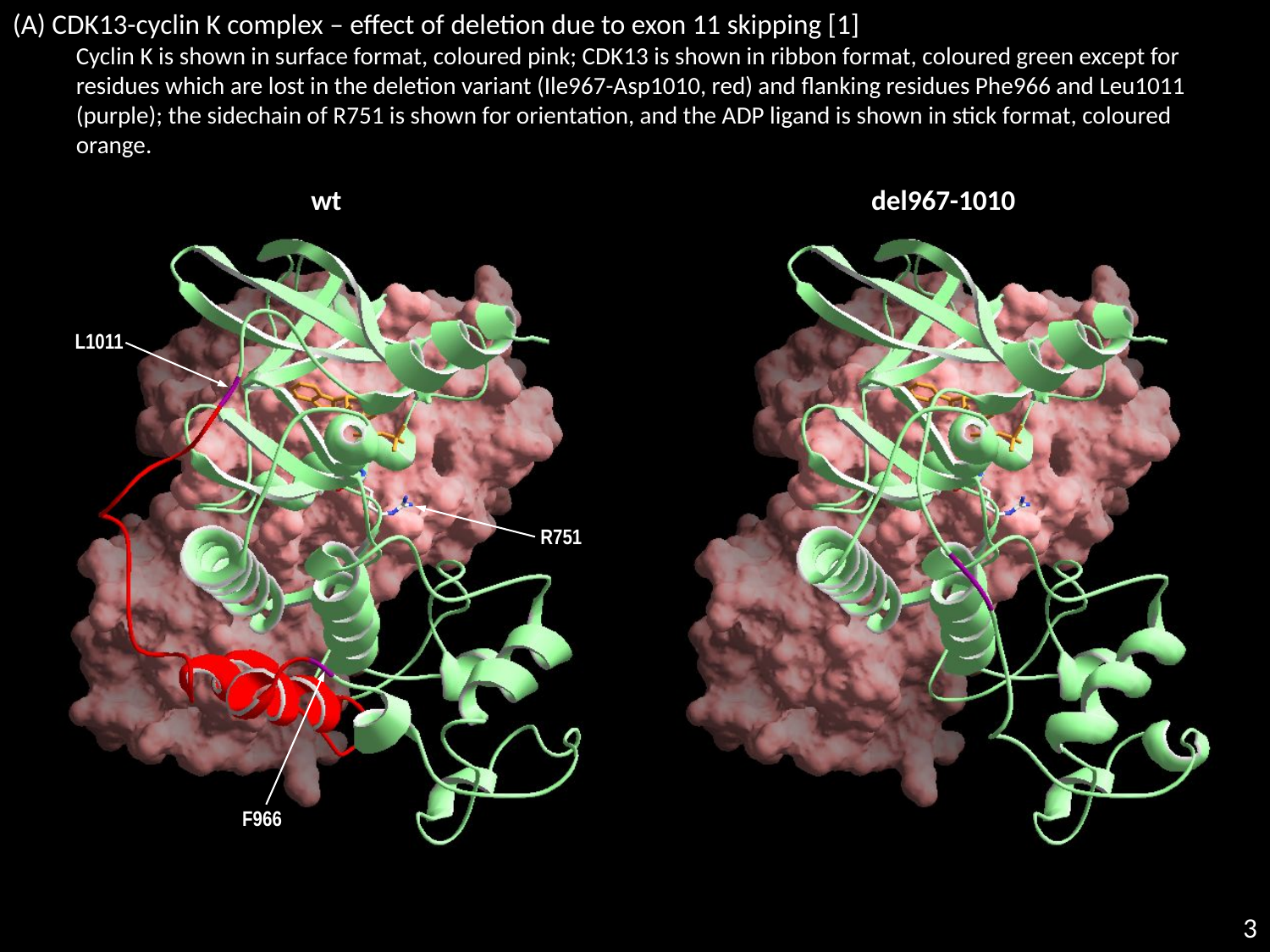

(A) CDK13-cyclin K complex – effect of deletion due to exon 11 skipping [1]
Cyclin K is shown in surface format, coloured pink; CDK13 is shown in ribbon format, coloured green except for residues which are lost in the deletion variant (Ile967-Asp1010, red) and flanking residues Phe966 and Leu1011 (purple); the sidechain of R751 is shown for orientation, and the ADP ligand is shown in stick format, coloured orange.
wt
del967-1010
L1011
R751
F966
3

## Slide 4
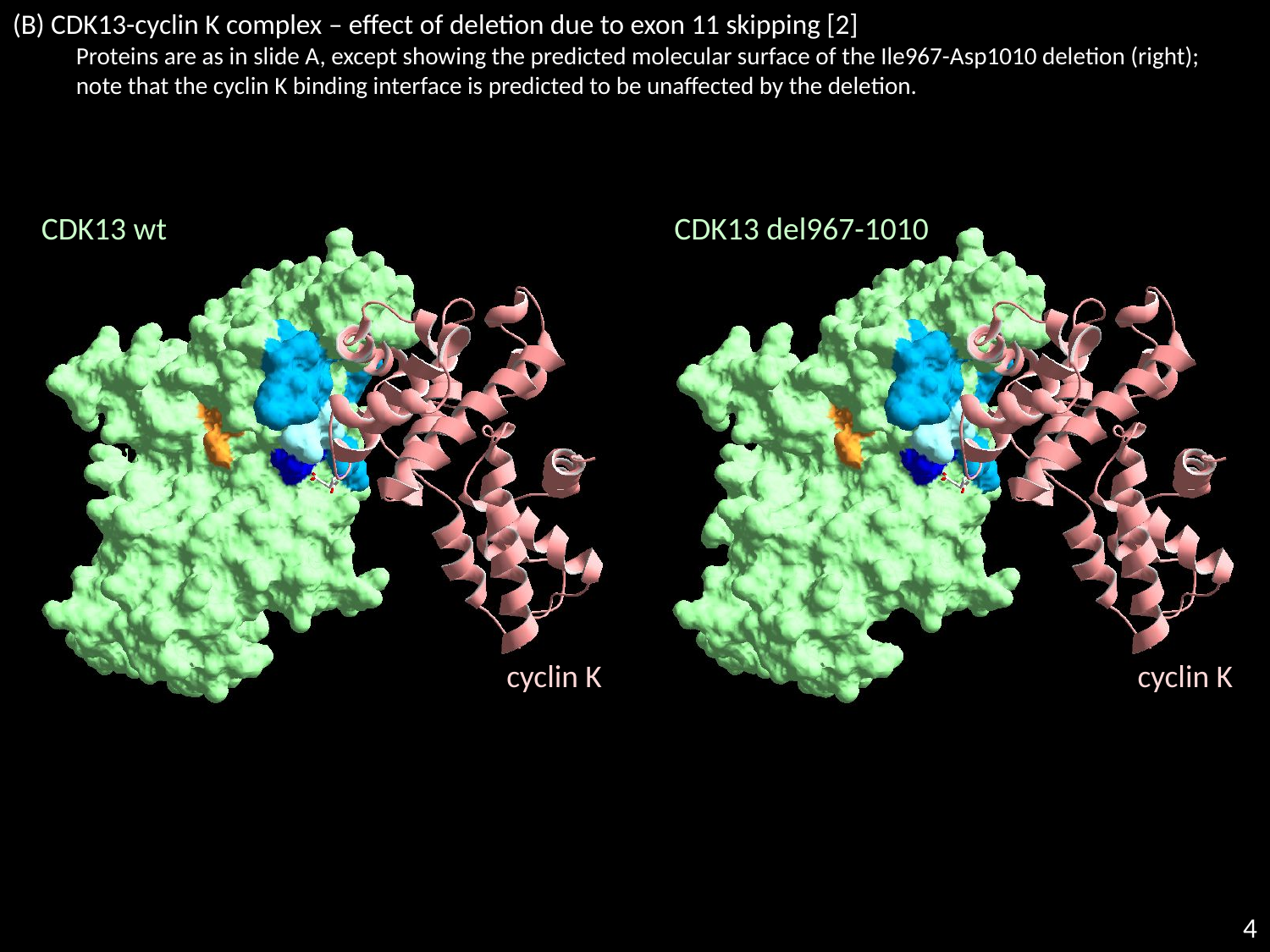

(B) CDK13-cyclin K complex – effect of deletion due to exon 11 skipping [2]
Proteins are as in slide A, except showing the predicted molecular surface of the Ile967-Asp1010 deletion (right); note that the cyclin K binding interface is predicted to be unaffected by the deletion.
CDK13 wt
CDK13 del967-1010
cyclin K
cyclin K
4
