# Supplementary material for: Heterozygous mutations affecting the protein kinase domain of CDK13 cause a syndromic form of developmental delay and intellectual disability
Source: J Med Genet. 2017 Oct 11;55(1):28–38. doi: 10.1136/jmedgenet-2017-104620 (PMC5749303; doi:10.1136/jmedgenet-2017-104620)
Supplement: Supplementary file 6 [file jmedgenet-2017-104620supp006.docx]

**S6 Supplementary table: Comparative modelling of novel missense variants – summary of effects**

| Gly714Arg | Novel Arg sidechain occludes ligand-binding pocket (Swiss-Model returns model without ligand) |
| --- | --- |
| Val719Gly | Sidechain of Val719 packs against that of Ile711 to stabilize upper face of ligand-binding pocket; glycine substitution expected to affect folding/structure of pocket (Swiss-Model returns model without ligand) |
| Lys734Arg | Swiss-Model returns model without ligand – manual construction of CDK13-ADP complex shows aberrant binding of arginine sidechain to ADP, and possible occlusion of binding pocket |
| Arg860Gln | Loss of salt bridge and H-bonding to phospho-Thr871; likely to result in loss of response to activating phosphorylation at Thr871 |
| Val874Leu | Sidechain of Val874 packs against others in interior of activation loop; packing is disrupted by larger sidechain of leucine, likely to destabilize folding/structure of this region |
| Asp896Asn | Sidechain of Asp896 forms hydrogen bonds to the backbone of His835 and Arg836, which in turn bond to Asp837 (the active site proton acceptor) and phospho-Thr871 (in the activation loop) respectively, thus providing a molecular link between the activation loop and the active site; in the Asp896Asn variant, H-bonding to Arg836 is lost, likely impairing the ability of CDK13 to respond to the activating phosphorylation at threonine 871 |
