# Supplementary material for: Heterozygous mutations affecting the protein kinase domain of CDK13 cause a syndromic form of developmental delay and intellectual disability
Source: J Med Genet. 2017 Oct 11;55(1):28–38. doi: 10.1136/jmedgenet-2017-104620 (PMC5749303; doi:10.1136/jmedgenet-2017-104620)
Supplement: Supplementary file 7 [file jmedgenet-2017-104620supp007.pptx]

## Slide 1
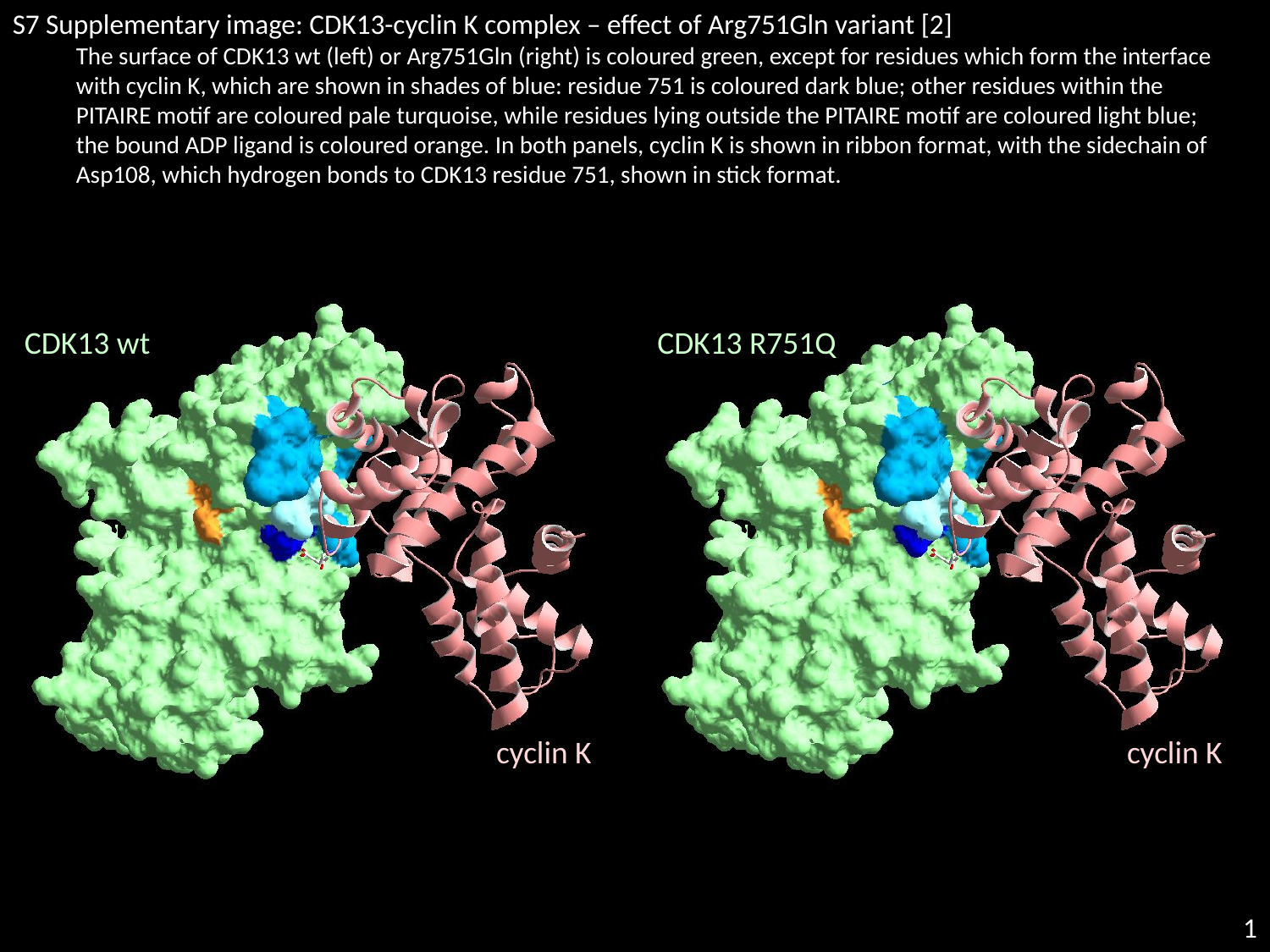

S7 Supplementary image: CDK13-cyclin K complex – effect of Arg751Gln variant [2]
The surface of CDK13 wt (left) or Arg751Gln (right) is coloured green, except for residues which form the interface with cyclin K, which are shown in shades of blue: residue 751 is coloured dark blue; other residues within the PITAIRE motif are coloured pale turquoise, while residues lying outside the PITAIRE motif are coloured light blue; the bound ADP ligand is coloured orange. In both panels, cyclin K is shown in ribbon format, with the sidechain of Asp108, which hydrogen bonds to CDK13 residue 751, shown in stick format.
CDK13 wt
CDK13 R751Q
cyclin K
cyclin K
1
